# Assessing the Association Between Animal Color and Behavior: A Meta‐Analysis of Experimental Studies

**DOI:** 10.1002/ece3.70655

**Published:** 2024-12-04

**Authors:** Sarah N. Ruckman, Eve A. Humphrey, Lily Muzzey, Ioanna Prantalou, Madison Pleasants, Kimberly A. Hughes

**Affiliations:** ^1^ Department of Biological Science Florida State University Tallahassee Florida USA; ^2^ Biology Department Lincoln University Pennsylvania USA

**Keywords:** aggressive behavior, badge of status, coloration, condition dependence, pleiotropy

## Abstract

Color varies in pattern and degree across the tree of life. In animals, genetic variation in color is hypothesized to have pleiotropic effects on a variety of behaviors due to shared dependence on underlying biochemical pathways. Such pleiotropy can constrain the independent evolution of color and behavior. Although associations between color and behavior have been reported, this relationship has not yet been addressed across a broad taxonomic scale with a formal meta‐analysis. We used a phylogenetic meta‐analytic approach to examine the relationship between individual variation in aggressive behavior and variation in multiple colors. Seventy‐four studies met our inclusion criteria (vertebrates = 70; invertebrates = 4). After accounting for phylogeny and correcting for publication bias, there was a positive association between measures of aggression and degree or area of coloration (mean = 0.248, 95% CI = (0.044, 0.477)). Because this positive association was not restricted to melanin‐based coloration, we conclude that this pattern does not strongly support the melanin pleiotropy hypothesis. Because the association was also not affected by moderators accounting for individual condition, social rank, or age, the results do not strongly support hypotheses that condition dependence accounts for relationships between color and aggressive behavior. The badge of status hypothesis predicts that arbitrary traits can evolve to signal aggression or social dominance. We propose that this is the most parsimonious explanation for the patterns we observe. Because of the lack of evidence for condition dependence in the association between color and aggression, we further propose that the genetic covariation between traits contributes to the evolution of the badges of status.

## Graphical abstract

In animals, genetic variation in color is hypothesized to have pleiotropic effects on a variety of behaviors due to shared dependence on underlying biochemical pathways. We used a phylogenetic meta‐analytic approach to examine the relationship between individual variation in aggressive behavior and variation in multiple colors. We propose that the moderate‐positive correlation between aggression and coloration across Animalia that we observed is underlain by genetic covariation between behavior and color traits that serve as badges of status.

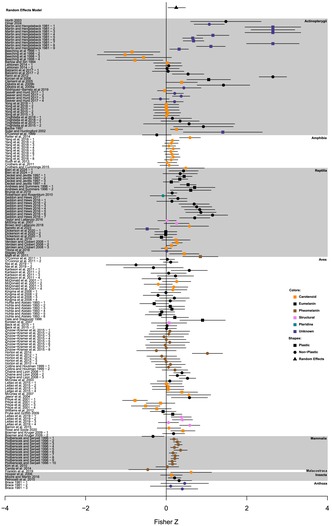

## 1 Introduction

A key goal of modern evolutionary biology is predicting if and how populations will evolve adaptively in response to environmental change. Predicting adaptive evolution requires knowing if there are constraints on how populations can respond to selection. The definition of adaptive constraint has been debated, but the unifying theme of these definitions is that populations are not always able to respond to selection in predictable ways (Blows and Walsh 2009; Walsh and Blows 2009).

A possible example of this kind of constraint has been widely discussed in both evolutionary and animal behavior literature. In animals, the biochemical pathways that regulate body coloration and behavior can overlap, potentially causing genetic covariance and imposing constraints on the joint evolution of color and behavior. In vertebrates, melanic coloration arises from the binding of melanocortin agonists to the G‐protein–coupled receptor MCR‐1. Melanocortins (peptide hormones derived from the prohormone proopiomelanocortin) also bind to other receptors that regulate diverse functions, including aggressive behavior (Ducrest, Keller, and Roulin 2008). Indeed, the literature summarized in Ducrest, Keller, and Roulin (2008; tables 1 and S2) and San‐Jose and Roulin (2018; table 1) indicates that the most widespread pattern is a positive correlation between intensity or extent of melanic coloration and high levels of aggression. Therefore, one prediction of this hypothesis is that heritable variation in abundance or activity of melanocortins generates genetic covariance between body color and aggression; animals with higher levels of eumelanic (dark brown and black) color are expected to exhibit higher levels of aggression (Ducrest, Keller, and Roulin 2008; Roulin and Ducrest 2011; San‐Jose and Roulin 2018). We note that other kinds of changes in the melanocortin system, such as mutation in melanocortin receptors that are expressed both in the skin and the brain, could also produce these correlations (e.g., Reissmann and Ludwig 2013). In support of this hypothesis, studies have reported correlations between melanin‐based coloration and behavior, including aggressive behavior, in vertebrates (e.g., Moore and Martin 2016; Dijkstra et al. 2017; Seddon and Hews 2017; Beck, Davies, and Sewall 2018). The hypothesis has recently been extended to include insects and other invertebrates (San‐Jose and Roulin 2018), where melanin is synthesized from dopamine. This invertebrate biosynthetic pathway provides a plausible link between body coloration and behavior (Wittkopp and Beldade 2009; Massey and Wittkopp 2016) and predicts correlations between traits that are similar to those seen in vertebrates. Indeed, similar correlations between traits have been reported in invertebrates (San‐Jose and Roulin 2018).

Other functional links between color and aggression have been hypothesized to arise from carotenoid condition dependence, whereby animals in good condition can devote more energy to high levels of aggression, and to carotenoid pigment synthesis, which is dependent on obtaining carotenoids in the diet (Blount and McGraw 2008; Backström et al. 2015). Under this scenario, genetic variants affecting foraging ability would simultaneously affect carotenoid color and aggressiveness, leading to genetic correlations between these traits. However, carotenoid condition dependence could also arise from purely environmental covariance between aggression and color if, for example, individual variation in foraging success arises from nongenetic variation (e.g., variation in habitat quality). Purely environmental covariance between color and aggression would not impose the same kind of adaptive constraint as would genetic covariance.

A third hypothesis is that a broad range of colors can be indicators or “badges” of social status. Badges of status are traits (e.g., color patches) that influence the outcome of aggressive encounters (Rohwer 1975; Dawkins and Krebs 1978; Diep and Westneat 2013). Badges of status are not limited to specific colors; badges can even lack color, as seen in the white forehead patch of collared flycatchers *Ficedula albicollis* (Pärt and Qvarnström 1997). A meta‐analysis of associations between dominance and plumage characteristics (color, UV presence, and color patch size) in birds reported a positive correlation between dominance and measures of coloration, irrespective of specific color, and the authors interpreted this result as supporting the badge of status hypothesis (Santos, Scheck, and Nakagawa 2011; but see Sánchez‐Tójar et al. 2018). A comparative analysis of competing bird species found that dominant species have on average more black than subordinate species; carotenoid and other colors were sometimes, but not always associated with dominance (Kenyon and Martin 2023). The authors interpreted this result as also supporting the badge of status hypothesis. Such badges can be honest signals (reliable predictors of aggressiveness), although the mechanism of maintaining honesty in the signal has been debated and could be different for each color or type of badge (Johnstone and Norris 1993; Tibbetts and Dale 2004). In ruffs, *Philomachus pugnax* , a supergene relates color and aggression, where the genes associated with color and behavior are located near each other and in linkage disequilibrium (Küpper et al. 2016; Lamichhaney et al. 2016). In contrast, some badges of status might not vary genetically, but might instead vary due to nongenetic sources. For example, some badges are plastic in expression and can vary as a result of dominance interactions (e.g., Dey, Dale, and Quinn 2014). As above, implications for adaptive constraint depend upon the underlying cause of trait covariance.

Other hypotheses can be consistent with a positive association between aggression and color, irrespective of the type of color. For example, melanin is endogenously produced; production and/or maintenance of melanic body color might be condition dependent, although the empirical evidence for this is mixed (Roulin 2016). Similar arguments can be made for structural colors. For example, if structural color depends on the condition of feathers or scales, then maintenance costs could be incurred in growing these structures and cleaning or removing ectoparasites from them. Structural color variation has been associated with conditions (e.g., McGraw et al. 2002) and mate quality (e.g., Siefferman and Hill 2003) in birds. If aggressive behavior is also condition dependent, then a general condition dependence might be responsible for positive associations between color and aggressive behavior, irrespective of type of color.

Understanding if and how color and behavior covary is critical to predicting evolutionary response to selection. This understanding is also essential for explaining why these ecologically important traits are often so highly variable among individuals, even within local populations (Roulin et al. 2008; Wellenreuther, Svensson, and Hansson 2014; Kraft et al. 2018; Santostefano et al. 2019). Constraint due to genetic covariance has mainly been discussed in relation to the melanocortin hypothesis, perhaps because melanin is less likely to be condition dependent than other pigment‐based colors (Ducrest, Keller, and Roulin 2008; Roulin and Ducrest 2011; San‐Jose and Roulin 2018). However, counterexamples of the expected association between darker color and higher aggression have been reported. Boerner and Krüger (2009) found that, in the common buzzard ( *Buteo buteo* ), light‐colored males are more aggressive than darker‐colored birds. In pied flycatchers ( *Ficedula hypoleuca* ), no significant relationship between male color and aggressive behavior was found (Huhta and Alatalo 1993). Examples such as these suggest that species (or populations within species) vary in the relationship between color and aggression. Counterexamples also raise the possibility that the preponderance of studies reporting significant correlations (Ducrest, Keller, and Roulin 2008; Roulin and Ducrest 2011; San‐Jose and Roulin 2018) reflects taxonomic or publication bias, or that correlations arise from specific features of studies like the age, sex, or condition of the focal animals. For example, a meta‐analysis reported a positive correlation between dominance and plumage traits in birds; this correlation was unaffected by the type of plumage trait but was influenced by the assessment method (whether aggression was assessed by quantifying specific aggressive acts, or by an indirect method such as distance between individuals; Santos, Scheck, and Nakagawa 2011). We know of no meta‐analyses of associations between coloration and behavior that extend across broader taxa, or that assess other factors such as whether color is fixed or plastic during adulthood.

Here, we describe a meta‐analysis of the (within‐species) relationship between body color variation and aggressive behavior across a broad taxonomic scale. The meta‐analysis included vertebrate and invertebrate taxa and controlled for effects of phylogeny on statistical inference. We investigated the possibility that publication bias has influenced the patterns reflected in the published literature. In addition, we evaluated whether the relationship between color and aggression is moderated by the type of color class (e.g., eumelanic, carotenoid, or structural), whether coloration is fixed or varies plastically during adulthood, life stage and sex of the focal animals, type of population studied (wild, domestic, lab‐reared, or wild caught and then lab tested), type of aggressive act measured (direct or indirectly measured), geographic origin of the species or source population of the focal animals, and whether or not social rank, age, and the condition of the animal were controlled or measured. We were especially interested in whether associations were moderated by color type because the melanocortin pleiotropy hypothesis predicts specifically that variation in eumelanin‐based colors should be associated with aggression. By contrast, condition dependence has most often been discussed in relation to carotenoid pigmentation (and predicts a positive association between carotenoid pigmentation and aggression). Consequently, positive associations between color and aggression that are restricted to melanic or carotenoid colors would provide direct support for those hypotheses. Both the badge of status hypothesis and general condition dependence predict that associations should be found between aggression and color, but that these associations should not be limited to melanin‐ or carotenoid‐based colors. The general condition‐dependence mechanism depends upon both color and aggression being condition dependent, leading us to predict that controlling for condition should moderate the relationship between color and behavior. By contrast, the badge of status hypothesis does not, a priori, make that same prediction. The badge of status hypothesis predicts that irrespective of color class and condition dependence, color should be positively associated with behavior (Rohwer 1975; Diep and Westneat 2013).

## 2 Methods

### 2.1 Data Collection

**FIGURE 1:**
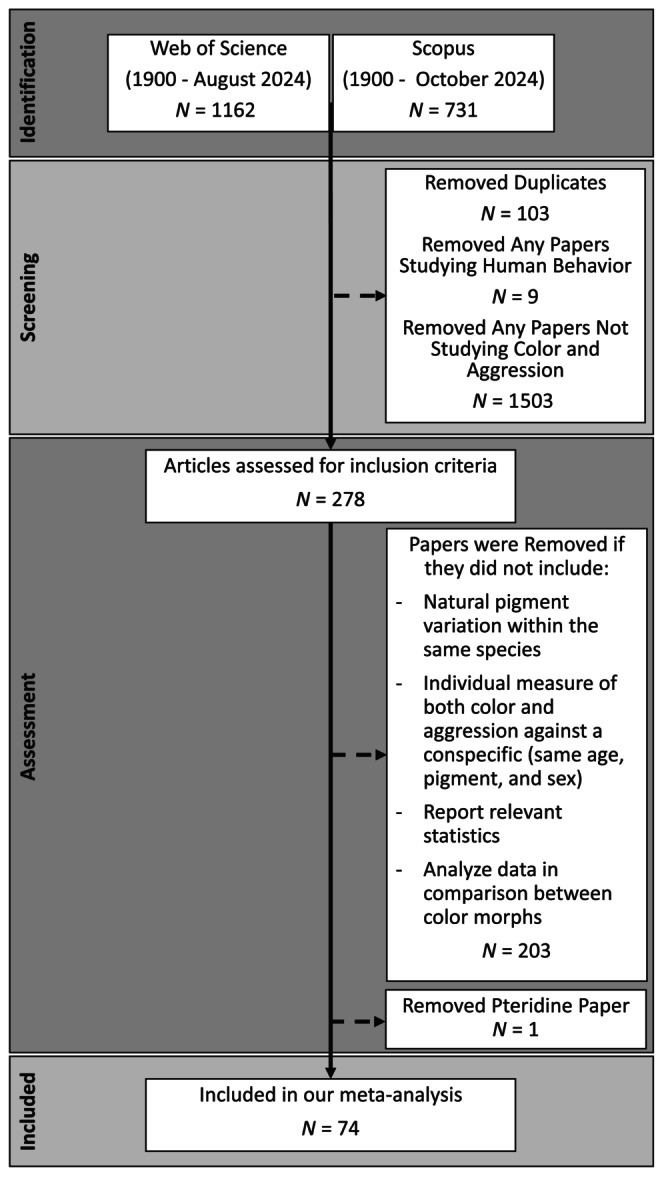
PRISMA diagram for papers examined. Solid arrows indicate papers that moved on to the next step and dashed arrows indicate papers that were removed from the analysis. We identified papers using Web of Science and Scopus (1162 and 731 papers, respectively). First, we removed any duplicate results from our search (*n* = 103). Next, we screened these papers for human studies and those that clearly did not examine color and aggression. This removed 1512 papers. Finally, we assessed the 278 papers based on our inclusion criteria and removed any studies that did not meet all the inclusion criteria. We removed one paper that fit our inclusion criteria because it was the sole paper on pteridine‐based coloration (Robertson and Rosenblum 2010). Seventy‐four papers met our inclusion criteria and were analyzed in our meta‐analysis.

We followed the reporting guidelines set out by O'Dea et al. (2021). We searched Web of Science using a basic search on all fields using the following terms (with lemmatization): aggression AND color OR color NOT cancer NOT oncology AND conspecific; each term was added with the Add Row button and the Boolean operator (Figure 1). We limited the search to Article (Document Type) under the refine results section. Finally, under the refine results section we chose the Web of Science categories: zoology, behavioral sciences, ecology, evolutionary biology, biology, ornithology, marine freshwater biology, entomology, fisheries, veterinary sciences, or agriculture dairy animal science. This yielded 1162 papers as of August 28, 2024. Results of this search are saved at: https://www.webofscience.com/wos/woscc/summary/2e26f841‐1b1f‐4cf2‐97c7‐a56132477b0c‐01195e86ad/relevance/1. In addition, we searched Scopus using the advanced search: (ALL(aggression) AND ALL(color OR color) AND ALL (evolution OR ecology OR behavior OR behavior) AND ALL(conspecific) AND NOT ALL (cancer) AND NOT ALL(oncology) AND NOT ALL (Medical) AND NOT ALL (medicine) AND NOT ALL (plant) AND NOT ALL (human) AND NOT ALL (psychology)). We also limited the Scopus search to the subject area of agricultural and biological sciences. This yielded 731 papers as of October 14, 2024. We then removed duplicates between the two searches (*n* = 103). Next, we removed studies focused on human subjects because the variation in human pigmentation is not due to the same mechanisms that have been proposed to lead to pleiotropic effects on behavior (Deng and Xu 2017; San‐Jose and Roulin 2018). This removed nine papers. We also removed any studies that did not measure both individual color and aggression (*n* = 1503), which is necessary to determine an association. This yielded 278 papers. We removed studies if they did not include any of the following: natural color variation within the same species, individual measures of both color and aggression against a conspecific (same age, sex, and color), and relevant statistics comparing color and aggression for effect size calculations. We removed one final paper that met our inclusion criteria because it was the sole paper focused on pteridine‐based pigmentation (Robertson and Rosenblum 2010). This yielded 74 papers (169 effect size estimates) that were included in the phylogenetic meta‐analysis.

We defined aggression as any variable that measured antagonistic behaviors (e.g., biting or chasing) toward a conspecific (of same sex, color class, and age class) or mirror image. We used only measures of aggression between individuals of the same sex because sexes can differ in both behavior and coloration as well as in how they respond to signals in other individuals (e.g., Horth 2003). Colors included those regulated by melanin‐ and carotenoid‐based pigments. We also included colors produced by structural variation in skin, scales, feathers, and cuticles (e.g., blue, purple, and white colors produced by iridophores in some fish; Schartl et al. 2016). Each color classification per species is listed and justified with citations in Table S1. Many color variable species exhibit discrete color morphs (e.g., “orange” and “blue”). These discrete morphs often associate with discrete behavioral/social categories (e.g., territorial vs. nonterritorial individuals), and the nature of aggressive interactions within a morph (e.g., territorial males) can differ substantially from those between morphs (e.g., Lank et al. 1995; Sinervo and Lively 1996). We therefore limited our data set to measures of aggression within color morphs. If both adults and nonadults were included in a study, we only used measures that compared individuals of the same life stage or age class.

From each paper, we collected the species name, color (how color was categorized or quantified in the original study (e.g., white vs. tan and area of eumelanic pigmentation)), location of the color (e.g., total body color or eye color), type of color class (e.g., eumelanin or pheomelanin, carotenoid, structural, pteridine, or unknown), and the measure of aggression used (e.g., direct aggression such as bites, or indirect such as proximity). We also collected the life stage (adult vs. juvenile) and sex of the focal individuals, location of the study (wild, lab‐reared, domesticated, and wild individuals measured in the lab), vertebrate or invertebrate taxa, plasticity of pigment expression (plastic or fixed), seasonality of pigment trait (year‐round, breeding, or non‐breeding), geographic location of focal or source population, if the condition of the animal was controlled and how (e.g., weight, length, or weight and length), if social rank was considered in the study design and how (e.g., uncontrolled, dummy used, isolated, or recording used), if the age of the animal was controlled and how (e.g., same age or covariate in analysis), and whether the study was an observational or experimental study. Finally, we recorded the measure of association between color and aggression (means/standard deviations of discrete groups, *t*‐, *F*‐, or *χ* ^*2*^‐test statistics with the associated *p*‐values and degrees of freedom, or a correlation coefficient), and the sample sizes for each measure of association.

### 2.2 Effect Size Calculations

For a standardized effect size, when possible, we used reported Pearson's correlation coefficients between color and aggression, which we then converted to Fisher *Z* statistics. The Fisher *Z* transformation is recommended to normalize the sampling distribution of correlation coefficient estimates when sample sizes are small and produce less biased results (Silver and Dunlap 1987; Berry and Mielke 2000). The Fisher *Z* transformation also widens the distribution around 0, which is useful because Markov Chain Monte Carlo (MCMC) has difficulty producing accurate estimates when the true value of the mean is very close to zero (Lipsey and Wilson 2001; Hadfield 2010).

When studies did not report correlation coefficients, we used reported F or *χ* ^*2*^ statistics and converted these into correlation coefficient estimates using methods described in Nakagawa et al. (2007) and Lipsey and Wilson (2001). When studies analyzed categorical data using a *t* test or reported only means and standard deviations for discrete groups, we could not calculate the product–moment correlation coefficient (Pearson's *r*) and instead calculated the biserial correlation coefficient (Jacobs and Viechtbauer 2017). The biserial correlation coefficient is comparable to the product–moment correlation and can therefore be used in the same meta‐analysis (Jacobs and Viechtbauer 2017). We then converted both Pearson's and biserial correlation estimates to Fisher *Z* using the R package *DescTools* and the command FisherZ (Signorell 2021). Biserial correlation coefficients are sometimes calculated to be greater than 1 or less than −1 (Pustejovsky 2014); values outside this range are undefined under the Fisher *Z* transformation (Fisher 1915; Silver and Dunlap 1987). This was the case with five positive values in our data set; these five positive values were reasonably close to 1, total range = −0.77 to 1.33. We therefore converted the positive values greater than 1 to 0.99, as appropriate, as recommended by Pustejovsky (2014).

### 2.3 Phylogenetic Meta‐Analytic Model

To account for the nonindependence of the data due to evolutionary history and the relationship between species, we constructed a phylogenetic tree of all the species (56 in total, 52 vertebrates, and 4 invertebrates across nine classes; Figure S1). We used an ultrametric tree that was fully resolved to the species level, which we obtained from TimeTree.org (Hedges, Dudley, and Kumar 2006; Kumar et al. 2017). We then rooted the tree with the anemone *Phymactis clematis* as the outgroup because it falls outside of Bilateria, to which all species in our data set belong. We used the R packages *ape, phytools*, and *TreeTools* to obtain the relatedness matrix, root the tree, change the edge lengths from 0 to 0.00001, and plot the tree (Paradis, Claude, and Strimmer 2004; Revell 2012; Smith and Wickham 2019).

We used the R package *MCMCglmm* to perform the meta‐analysis (Hadfield 2010). We chose an expanded prior with a Cauchy distribution that mirrored the Fisher *Z* distribution (Adams 2008). We ran each model for 2,000,000 interactions and removed 1,000,000 steps as the burnin, and with a thin (the *i*th value kept in a run to reduce autocorrelation) of 1,000. Once we determined the models with the lowest DIC values, we reran the analysis using 5,000,000 iterations with burnin of 2,500,000 and a thin value of 1000; these parameters always produced convergence and final values that were in the Fisher *Z* distribution bounded by [−2.64, 2.64]. All credible intervals reported are based on the last 2,500,000 iterations of the *MCMCglmm*.

### 2.4 Random and Mixed Models

We first evaluated a model containing only random effects of species, study, the weights associated with each study (calculated as the inverse of the standard error for the Fisher *Z* for each study), and the phylogenetic tree. Weights were added as a variance–covariance matrix using the “us” option. Phylogenetic information was incorporated as a relatedness matrix with the “pedigree” option, which assumes a Brownian motion model of evolution (Hadfield 2010; Nakagawa and Santos 2012). We removed the random effects using backward elimination to confirm the importance of each random effect. We found that the model with the lowest DIC value was that which included all random effects (see Results). Retaining all random effects, we then assessed whether any moderators improved model fit compared to the random‐effects–only model (hereafter, “random effects model”). We tested each of the following moderators one at a time, and in all two‐way combinations: type of color classification, plasticity, sex, life stage, vertebrate or invertebrate, location of the study, seasonality of the color, geographic location of the study population, observational versus experimental studies, if condition of the animal was controlled and how, if social rank was controlled and how, if age of focal animals was controlled and how, and type of aggressive acts (direct or indirect). We also tested for two‐way interactions between pairs of moderators that we deemed biologically likely: color class by plasticity, color class by sex, and sex by plasticity. We compared these models using DIC values. For the best‐fitting models, we computed medians and 95% confidence intervals of the effect size for each moderator using the R package *emmeans* (Lenth et al. 2021).

Some of the moderators listed above were not available in all the studies in our data set: condition, social rank, and age of focal animals. We accounted for this in two ways. First, as described above, we used a moderator that indicated whether the feature was controlled for or used as a covariate in the study. For example, social rank could be uncontrolled, controlled by using unfamiliar animals, or controlled by using a mirror test or video. For each of these three moderators, we also asked if including only those studies that controlled for the moderator produced substantially different results. If the association between color and aggression were to vary with color type when including these moderators, that would suggest that condition dependence underlies associations for some colors, but not others. That is, these analyses were conducted to assess the possibility that associations of aggression with different color classes have different underlying mechanisms. Because we were particularly interested in any difference in effect size between different color classes, we always included color class as an additional moderator in these “subset” models.

Finally, we investigated whether effect sizes that were reported for “unknown” color classes were potentially modulated by the melanocortin pathway. Recent studies suggest that, at least in fish, the melanocortin system can regulate nonmelanin–based colors (reviewed in Cal et al. 2017). To determine if expanding the color classes regulated by the melanocortin system affected the results, we recoded all unknown color classes as eumelanin and reran the mixed‐effects model with color class as a moderator.

### 2.5 Heterogeneity

Ecology and evolutionary biology meta‐analyses are likely to exhibit high levels of heterogeneity due to differences in taxa and experimental methods across studies (Gurevitch and Hedges 1999; Senior et al. 2016). We used a method developed by Nakagawa and Santos (2012) to quantify the proportion of the total variance due to phylogeny (termed “phylogenetic signal” or *H* ^2^) in their equation 26, and the heterogeneity due to study ($I_{s}^{2}$) and species ($I_{a}^{2}$) using equations 24 and 25 of that paper, respectively. To make the values more easily interpretable, we reported the heterogeneity values as percentages, rather than proportions. Percentages close to 0 indicate low heterogeneity while percentages close to 100 are considered high heterogeneity. The phylogenetic signal was left as a proportion.

### 2.6 Publication Bias

Publication bias due to selective reporting of results can influence both the estimated magnitude and reliability of the overall effect size estimate (Rosenthal 1979; Gurevitch and Hedges 1999). We investigated the possibility of publication bias by visualizing study asymmetry using contour‐enhanced funnel plots and by testing for asymmetry using a modified Egger's test as described in Nakagawa and Santos (2012). This test regresses the meta‐analytic residuals on the precision of each effect size estimate (inverse of standard error). These residuals, unlike the weighted effect sizes themselves, are independent and thus satisfy the assumptions of the test. We corrected the effect of publication bias using the trim‐and‐fill method implemented in the R package *meta* (Duval and Tweedie 2000; Schwarzer 2007). This method restores funnel plot symmetry by iteratively removing studies with large positive residuals and imputing missing effect size estimates. Because we used meta‐analytic residuals for this analysis, the resulting estimates of the mean effect size estimates are the adjustments required to restore the funnel plot symmetry (Duval and Tweedie 2000). All analyses were conducted in R version 4.3.1 (R Core Team 2023). Data and scripts for all analyses are available in a GitHub repository (https://github.com/sruckman/meta‐analysis).

## 3 Results

### 3.1 Full Data Set Analysis

**FIGURE 2:**
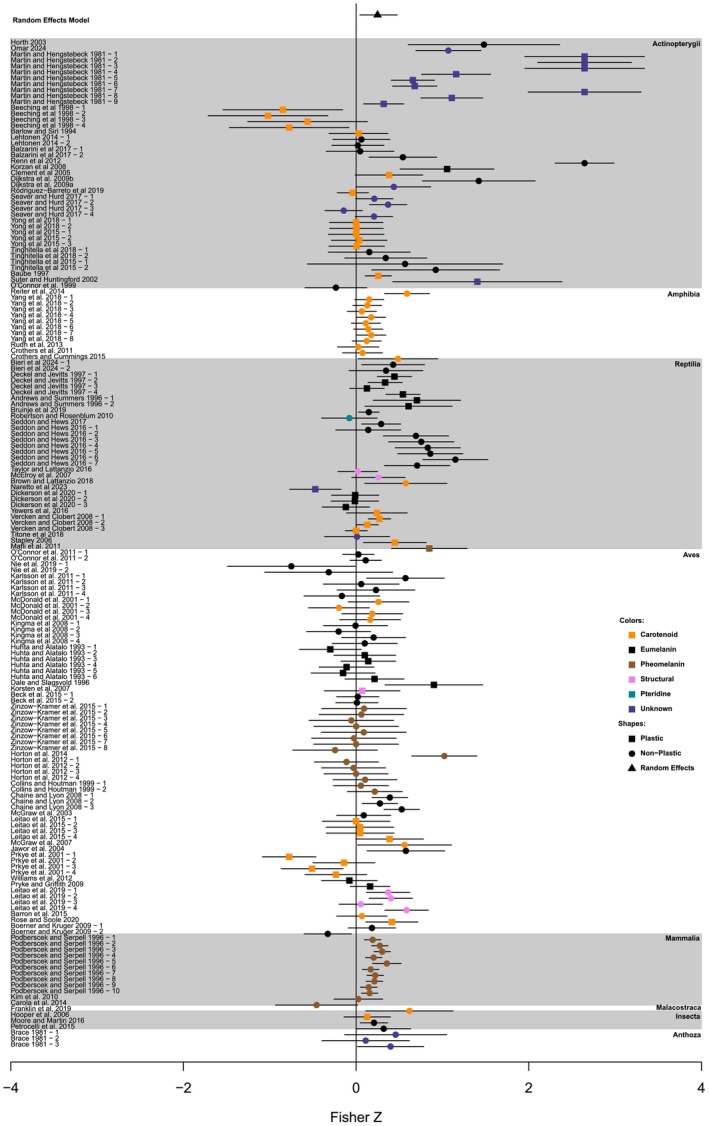
The effect size and 95% credible intervals for all studies listed by family. The study names are on the left side of the graph and any study with a dash then a number is a second effect size from the same study. The colors of the points indicate the color class, while the shape of the point indicates if the trait is plastic or non‐plastic. The points at the top of the graph are the mean effect size and 95% credible intervals for the random‐effects model. The black triangle and line through it indicate the model mean with 95% credible intervals.

We calculated 169 effect sizes from 74 studies that met our inclusion criteria. The random‐effects model with the lowest DIC value (−283.425) included all random effects (phylogenetic tree, species, study, and weights; Table S2). This model had a mean posterior effect size of 0.248 (95% credible interval = (0.044, 0.477); mean Pearson's correlation = 0.243 and 95% credible interval = (0.044, 0.444)), indicating support for a positive association between the intensity of color and aggression (Figure 2).

In the model that included all the random effects, phylogenetic signal accounted for 21.1% of the variation in the data set (Table S2, Figure 2). However, when we removed the phylogeny, species, and study effects sequentially, the only term that caused substantially poorer fit when removed was the study effects (Spiegelhalter et al. 2002; Table S2). Removing only the phylogenetic signal or removing the tree alone did not result in a poorer fit when compared to the full random‐effects model. This suggests that the “phylogenetic” signal arises because some taxonomic groups are represented by one or a very few studies (e.g., Amphibia, see Figure 2). We therefore interpret this signal as mainly reflecting variation among studies, not a true phylogenetic pattern.

None of the fixed‐effect moderators we investigated improved the fit of the full random‐effects model; that is, adding any moderator increased the DIC value. Some of the mixed‐effects models exhibited DIC values that were close to that of the full random‐effects model (Tables S2 and S3). For example, a mixed model that included type of aggressive act as a moderator had a DIC value that was only slightly larger than that of the full random‐effects model (−282.652, Table S3). No other model had a lower DIC value than these 2. Critically, no models that included color class (eumelanin, pheomelanin, carotenoid, structural, or unknown colors) as a moderator, either alone or in combination with any other moderator, ever achieved a DIC value less than −280 (i.e., larger than the random‐effects–only model by at least 3). Spiegelhalter et al. (2002) suggest that alternative models with DIC values “within 1–2 of the ‘best’ deserve consideration, and 3–7 have considerably less support.” Consequently, there is little support in this data set for color‐type differences in the association between intensity of color and aggression.

**FIGURE 3:**
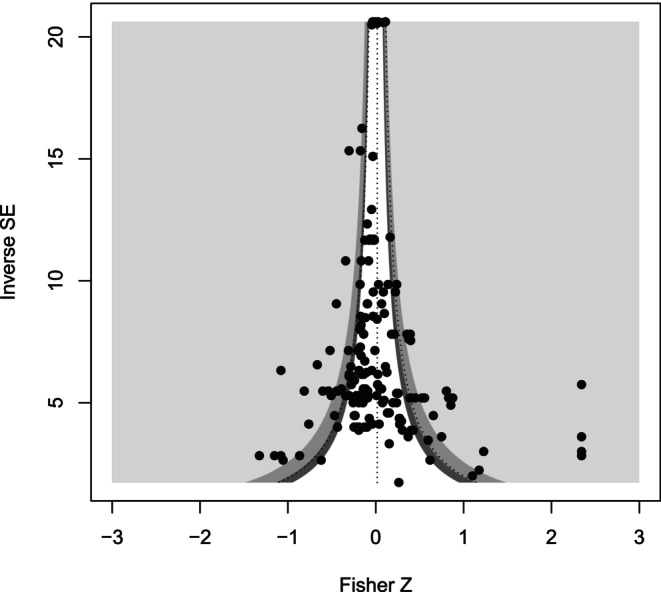
Funnel plot of random‐effects–only model. We found no evidence of a publication bias. Therefore, no points were needed to restore plot symmetry.

We did not find evidence of a publication bias for the full random‐effects model. In this model, the intercept for the modified Egger's regression was not significantly different from zero (intercept ± SE: 0.703 ± 0.372, *t*‐value = 1.89, *p*‐value = 0.0609). The trim‐and‐fill method added zero imputed values to the original 169 effect size estimates (Figure 3).

To determine if the melanocortin pleiotropy hypothesis would be better supported if we reclassified “unknown” color types as “eumelanic” (8 studies, 21 effect sizes), we ran the mixed‐effects model that included color class as a moderator on this recoded data set. This “recoded” mixed model had a similar posterior effect distribution (mean = 0.260, 95% credible interval: −0.012, 0.545). As in the original data set, adding the color moderator did not improve the fit (DIC value for recoded color model: −279.590, Table S3). Notably, the mixed model did not provide a better fit than the random‐effects model for either data set, suggesting that the relationship between color intensity and aggression does not vary based on the type of color, as predicted by the melanocortin pleiotropy hypothesis.

### 3.2 Analysis of Data Subsets

Results did not differ substantially in analyses of data subsets that included only those studies that controlled for animal condition (59 studies, 112 effect sizes), social rank (58 studies, 133 effect sizes), or age (16 studies, 25 effect sizes). As in analyses of the full data set, the random‐effects model was a better fit than a model that included color type as a moderator for every data subset (Table S4). The mean posterior effect sizes for the relationship between color and aggression were also similar in magnitude to the mean estimate for the full data set, ranging from 0.115 to 0.289 (Table S4). Fewer studies controlled for animal age (beyond juvenile/adult) compared to those that controlled for social rank or condition. Analysis of the subset that did control for age (16 studies) or for age, condition, and social rank (9 studies) produced similar mean posterior effect size estimates, but in these cases, the credible interval for the mean‐effect size overlapped zero (Table S4).

## 4 Discussion

The meta‐analysis of 74 published studies indicated a positive association between measures of aggression and measures indicating more colorful individuals. For simplicity, we used the term “colorful” to refer to variation in hue, intensity, or area as used in the original studies. No moderator that we evaluated improved the fit of the meta‐analytic model to the data, indicating a robust relationship between color and aggression. Critically, we found no evidence that this relationship depended on the type of color (eumelanic, phaeomelanic, carotenoid, or structural), counter to predictions of the melanin pleiotropy and carotenoid condition‐dependence hypotheses. This result was unchanged when we recoded colors of unknown cause, but for which a melanocortin‐based mechanism is plausible. Both the badge of status and general condition‐dependence hypotheses are consistent with this pattern. However, under general condition dependence, we expect the effect size to be sensitive to moderators indicating if and how condition was accounted for in the study design, or to moderators that are plausibly associated with condition, such as social rank and age. None of these moderators explained variation in effect sizes in our analyses, and none affected the (lack of) dependence of effect size on type of color.

It is possible that different mechanisms underlie similar correlations between aggression and different kinds of coloration. For example, it is possible that covariation between melanin‐based colors and aggression in the studies is indeed regulated by variation in the melanocortin pathway, and that covariation between aggression and carotenoid color is regulated by condition dependence that was not accounted for by any of the proxies for condition that we analyzed. However, the badge of status hypothesis is a more parsimonious explanation for these patterns (Tibbetts and Dale 2004; Santos, Scheck, and Nakagawa 2011).

A recent analysis of the badge of status hypothesis in house sparrows concluded that evidence for a positive association between black throat patches and aggression was equivocal (Sánchez‐Tójar et al. 2018). However, the mean effect size in their primary meta‐analysis (0.23, with 95% credible interval [−0.01, 0.45]) is very close to the value we found across all taxa (0.25, 95% credible interval [0.04, 0.4]). Our analysis included more studies and more effect sizes, which could account for our somewhat narrower credible interval. Nevertheless, the two estimates are remarkably consistent given the difference in taxonomic scale and sample size.

For the studies included in our data set, a wide variety of metrics of color were used, typically reflecting the natural variation observed in the focal species. It is conceivable that patterns of covariance might depend on the specific type of variation in color that is contrasted. For example, variation in intensity of melanic coloration within a given area might be more (or less) likely to be influenced by the “melanocortin pleiotropy” mechanism. Quantification and reporting of multiple aspects of color would enable testing of this idea, but very few studies we surveyed included such metrics (four studies).

It is conceivable that failure of moderators to improve the fit of the model is a result of including studies with low sample size and high variation, or of including few studies assessing the moderators of interest (Ginzburg and Jensen 2004; Lajeunesse 2009). However, for key moderators, our analysis had robust sample sizes. For example, in the condition category, 112 measures were derived from studies that accounted for animal conditions in some way, while 57 measures did not. In the subset of data that included only studies accounting for condition in some way, 47 measures accounted for both length and weight, 13 for weight only, and 52 for length only. In addition, the low heterogeneity observed in the pure random‐effects model suggests that power was not severely compromised by low sample size or high uncertainty in effect size estimates. Nevertheless, some combinations of moderators (e.g., color class, social rank, and age) were represented by few effect sizes in our data set (Table S4). Consequently, if condition is affected by adult age, as seems likely, current literature might be inadequate to evaluate the general condition dependence hypothesis. In addition, only a handful of studies of invertebrates met our inclusion criteria. We therefore urge caution in interpreting the lack of taxonomic effect at this level.

We did observe variation in effect sizes among studies, and among‐study variation was the main contributor to heterogeneity in our analysis. In contrast, species and phylogenetic relatedness did not explain additional heterogeneity. For example, the most extreme effect sizes (both positive and negative) are found within Actinopterygii (Figure 2). The strongest negative association was derived from a single study of orange color intensity in female convict cichlids (Beeching et al. 1998). In that study, females with the most orange color displayed the lowest level of aggression toward a stimulus female. The most positive associations between color and aggression occurred in a study of plastic eye color in juvenile guppies ( *Poecilia reticulata* ; Martin and Hengstebeck 1981). Fish with darker eye color engaged in and won more aggressive encounters than the light‐eyed fish. Losers in these encounters also lightened their eye color, consistent with some studies of traits deemed to be badges of status (Dey, Dale, and Quinn 2014).

In our data set, effect sizes within studies tended to be tightly clustered. This pattern could be driven by differences in methodology across studies, as was found in a meta‐analysis of birds (Santos, Scheck, and Nakagawa 2011). However, we categorized studies based on whether measures of aggression were direct or indirect (as in Santos, Scheck, and Nakagawa 2011) and based on whether the general methodology was observational or experimental. Neither of these categorizations was associated with variation in effect size.

Of the four hypotheses explaining consistent associations between animal coloration and aggression, our results are most parsimoniously explained by the badge of status hypothesis. This hypothesis proposes that aggressiveness, fighting ability, or dominance status is honestly reflected by variation in a trait that is perceptible to conspecifics (Rohwer 1975; McGraw, Dale, and Mackillop 2003; Tibbetts and Dale 2004). While many studies suggest that melanocortin‐based genetic pleiotropy can constrain the joint evolution of color and behavior, whether the badge of status mechanism should impose such constraints has received less attention. Under the badge of status hypothesis, the correlation between color and aggression could be caused by pleiotropy or tight linkage (Santos, Scheck, and Nakagawa 2011; Küpper et al. 2016; Lamichhaney et al. 2016; Sánchez‐Tójar et al. 2018). The supergene regulating feather coloration and social status in ruffs is a good example (Küpper et al. 2016; Lamichhaney et al. 2016). The relationship between head stripe color and aggression in white‐throated sparrows is another likely example of genetically based covariation that arises from the badge of status mechanism (Lowther 1961; Knapton and Falls 1983; Tuttle 2003; Tuttle et al. 2016; Hedrick, Tuttle, and Gonser 2018). By contrast, variation in a badge of status could also be regulated by nongenetic variation in resource availability or acquisition, variation in exposure to disease or parasites, or nongenetic maternal effects (Rohwer 1975; Dawkins and Krebs 1978). In that case, however, we would expect controlling for condition to moderate the association between color and aggression, but we do not see that. We, therefore, propose that the moderate correlation between color and aggression is underlain by genetic covariation between behavior and color traits that serve as badges of status.

To predict response to environmental change and to understand why ecologically important traits like color and behavior are so variable within populations, we need to know if these traits consistently covary in animal populations and if that covariation could constrain evolutionary response to changing environments. Given (1) the seemingly robust association we detect and (2) the relatively modest size of that effect, what conclusions can we draw about the level of evolutionary constraint imposed by color–behavior associations in animals? We know that even moderate genetic correlations can constrain response to selection (Charlesworth 1990; Houle 1991; Walsh and Blows 2009). However, for any given estimate of correlation, it is difficult to predict if it will substantially constrain adaptive evolution. The hypothesis that color–behavior associations in animals do constrain adaptive evolution could be tested directly in some organisms (mainly short‐lived invertebrates) using artificial selection or experimental evolution. In addition, several long‐term studies of free‐living organisms (mainly vertebrates) now have pedigree and phenotypic data that could allow testing this hypothesis (Clutton‐Brock and Pemberton 2004; Blondel et al. 2006; Charmantier et al. 2006; Foerster et al. 2007; McAdam et al. 2007). These kinds of data will become increasingly available as more long‐term studies incorporate genomic data. Consequently, both experimental and nonexperimental studies can expand our understanding of constraints imposed by color–behavior associations.

## Author Contributions

**Sarah N. Ruckman:** conceptualization (equal), data curation (lead), formal analysis (lead), investigation (lead), methodology (lead), project administration (equal), supervision (equal), validation (equal), visualization (equal), writing – original draft (equal), writing – review and editing (equal). **Eve A. Humphrey:** data curation (supporting), investigation (supporting), methodology (supporting), project administration (equal), writing – original draft (equal), writing – review and editing (equal). **Lily Muzzey:** data curation (equal), investigation (equal). **Ioanna Prantalou:** data curation (equal), investigation (equal). **Madison Pleasants:** data curation (equal), investigation (equal). **Kimberly A. Hughes:** conceptualization (equal), formal analysis (supporting), funding acquisition (lead), project administration (equal), supervision (equal), validation (equal), writing – original draft (equal), writing – review and editing (equal).

## Conflicts of Interest

The authors declare no conflicts of interest.

## Supporting information


**Figure S1.** Phylogeny for papers examined. The phylogeny is an ultrametric tree that was fully resolved to the species level, which we obtained from TimeTree.org.

**Table S1.** Body color classification justification. Every species included in the meta‐analysis is listed, along with categories of the colors used by the study authors (Columns 1–3). Justification for the classification of the colors used in the meta‐analysis is in Columns 6–7.

**Table S2.** Table of model DIC values for random‐effects models.

**Table S3.** Table of Model DIC values for mixed‐effects models. All means and 95% credible intervals are in correlation coefficient values rather than Fisher *Z* values for interpretability.

**Table S4.** Table of model DIC values for subset models. Random‐effects–only models of different data subsets are listed first in the table, followed by corresponding models that included the color moderator. All means and 95% credible intervals are correlation coefficients rather than Fisher *Z* values and are corrected for publication bias when necessary.

## Data Availability

All data and scripts are available online at https://github.com/sruckman/meta‐analysis. Data are publicly available via the DRYAD repository at https://doi.org/10.5061/dryad.9kd51c5tk.

## References

[ece370655-bib-0001] Adams, D. C. 2008. “Phylogenetic Meta‐Analysis.” Evolution 62: 567–572. 18182073 10.1111/j.1558-5646.2007.00314.x

[ece370655-bib-0002] Andrews, T. J. , and C. H. Summers . 1996. Aggression, and the Acquisition and Function of Social Dominance in Female *Anolis carolinensis* . Brill. 10.1163/156853996X00396.

[ece370655-bib-0003] Backström, T. , M. Heynen , E. Brännäs , J. Nilsson , and C. Magnhagen . 2015. “Dominance and Stress Signalling of Carotenoid Pigmentation in Arctic Charr ( *Salvelinus alpinus* ): Lateralization Effects?” Physiology & Behavior 138: 52–57. 25447479 10.1016/j.physbeh.2014.10.003

[ece370655-bib-0004] Balzarini, V. , M. Taborsky , F. Villa , and J. G. Frommen . 2017. “Computer Animations of Color Markings Reveal the Function of Visual Threat Signals in *Neolamprologus pulcher* .” Current Zoology 63: 45–54. 29491962 10.1093/cz/zow086 PMC5804153

[ece370655-bib-0005] Barlow, G. W. , and P. Siri . 1994. “Polychromatic Midas Cichlids Respond to Dummy Opponents: Color, Contrast and Context.” Behaviour 130: 77–112.

[ece370655-bib-0006] Barron, D. G. , M. S. Webster , and H. Schwabl . 2015. “Do Androgens Link Morphology and Behaviour to Produce Phenotype‐Specific Behavioural Strategies?” Animal Behaviour 100: 116–124.

[ece370655-bib-0007] Baube, C. L. 1997. “Manipulations of Signalling Environment Affect Male Competitive Success in Three‐Spined Sticklebacks.” Animal Behaviour 53: 819–833.

[ece370655-bib-0008] Beck, M. L. , S. Davies , and K. B. Sewall . 2018. “Urbanization Alters the Relationship Between Coloration and Territorial Aggression, but Not Hormones, in Song Sparrows.” Animal Behaviour 142: 119–128.

[ece370655-bib-0009] Beeching, S. C. , S. H. Gross , H. S. Bretz , and E. Hariatis . 1998. “Sexual Dichromatism in Convict Cichlids: The Ethological Significance of Female Ventral Coloration.” Animal Behaviour 56: 1021–1026. 9790714 10.1006/anbe.1998.0868

[ece370655-bib-0010] Berry, K. J. , and P. W. Mielke . 2000. “A Monte Carlo Investigation of the Fisher Z Transformation for Normal and Nonnormal Distributions.” Psychological Reports 87: 1101–1114. 11272750 10.2466/pr0.2000.87.3f.1101

[ece370655-bib-0011] Bieri, E. , A. O. Rubio , and K. Summers . 2024. “Beyond Color and Pattern: Elucidating the Factors Associated With Intraspecific Aggression in the Mimic Poison Frog ( *Ranitomeya imitator* ).” Evolutionary Ecology 38: 621–638. 10.1007/s10682-023-10285-x.

[ece370655-bib-0012] Blondel, J. , D. W. Thomas , A. Charmantier , P. Perret , P. Bourgault , and M. M. Lambrechts . 2006. “A Thirty‐Year Study of Phenotypic and Genetic Variation of Blue Tits in Mediterranean Habitat Mosaics.” Bioscience 56: 661–673.

[ece370655-bib-0013] Blount, J. D. , and K. J. McGraw . 2008. “Signal Functions of Carotenoid Colouration.” In Carotenoids: Volume 4: Natural Functions, edited by G. Britton , S. Liaaen‐Jensen , and H. Pfander , 213–236. Basel: Birkhäuser.

[ece370655-bib-0014] Blows, M. , and B. Walsh . 2009. “Spherical Cows Grazing in Flatland: Constraints to Selection and Adaptation.” In Adaptation and Fitness in Animal Populations: Evolutionary and Breeding Perspectives on Genetic Resource Management, edited by J. van der Werf , H.‐U. Graser , R. Frankham , and C. Gondro , 83–101. Netherlands, Dordrecht: Springer.

[ece370655-bib-0015] Boerner, M. , and O. Krüger . 2009. “Aggression and Fitness Differences Between Plumage Morphs in the Common Buzzard ( *Buteo buteo* ).” Behavioral Ecology 20: 180–185.

[ece370655-bib-0016] Brace, R. C. 1981. “Intraspecific Aggression in the Colour Morphs of the Anemone *Phymactis clematis* From Chile.” Marine Biology 64: 85–93.

[ece370655-bib-0017] Brown, D. M. , and M. S. Lattanzio . 2018. “Resource Variability and the Collapse of a Dominance Hierarchy in a Colour Polymorphic Species.” Behaviour 155: 443–463.

[ece370655-bib-0018] Cal, L. , P. Suarez‐Bregua , J. M. Cerdá‐Reverter , I. Braasch , and J. Rotllant . 2017. “Fish Pigmentation and the Melanocortin System.” Comparative Biochemistry and Physiology. Part A, Molecular & Integrative Physiology 211: 26–33. 10.1016/j.cbpa.2017.06.001 28599948

[ece370655-bib-0019] Carola, V. , E. Perlas , F. Zonfrillo , H. A. Soini , M. V. Novotny , and C. T. Gross . 2014. “Modulation of Social Behavior by the Agouti Pigmentation Gene.” Frontiers in Behavioral Neuroscience 8: 259. 25136298 10.3389/fnbeh.2014.00259 PMC4117936

[ece370655-bib-0020] Chaine, A. S. , and B. E. Lyon . 2008. “Intrasexual Selection on Multiple Plumage Ornaments in the Lark Bunting.” Animal Behaviour 76: 657–667.

[ece370655-bib-0021] Charlesworth, B. 1990. “Optimization Models, Quantitative Genetics, and Mutation.” Evolution 44: 520–538. 28567983 10.1111/j.1558-5646.1990.tb05936.x

[ece370655-bib-0022] Charmantier, A. , C. Perrins , R. H. McCleery , and B. C. Sheldon . 2006. “Evolutionary Response to Selection on Clutch Size in a Long‐Term Study of the Mute Swan.” American Naturalist 167: 453–465. 10.1086/499378 16673352

[ece370655-bib-0023] Clement, T. S. , V. Parikh , M. Schrumpf , and R. D. Fernald . 2005. “Behavioral Coping Strategies in a Cichlid Fish: The Role of Social Status and Acute Stress Response in Direct and Displaced Aggression.” Hormones and Behavior 47: 336–342. 15708763 10.1016/j.yhbeh.2004.11.014

[ece370655-bib-0024] Clutton‐Brock, T. H. , and J. M. Pemberton , eds. 2004. Soay Sheep: Dynamics and Selection in an Island Population. Cambridge UK; New York: Cambridge University Press.

[ece370655-bib-0025] Collins, C. E. , and A. M. Houtman . 1999. “Tan and White Color Morphs of White‐Throated Sparrows Differ in Their Non‐Song Vocal Responses to Territorial Intrusion.” Condor 101: 842–845.

[ece370655-bib-0026] Crothers, L. , E. Gering , and M. Cummings . 2011. “Aposematic Signal Variation Predicts Male–Male Interactions In A Polymorphic Poison Frog.” Evolution 65: 599–605. 21271999 10.1111/j.1558-5646.2010.01154.x

[ece370655-bib-0027] Crothers, L. R. , and M. E. Cummings . 2015. “A Multifunctional Warning Signal Behaves as an Agonistic Status Signal in a Poison Frog.” Behavioral Ecology 26: 560–568.

[ece370655-bib-0028] Dale, S. , and T. Slagsvold . 1996. “Mate Choice on Multiple Cues, Decision Rules and Sampling Strategies in Female Pied Flycatchers.” Behaviour 133: 903–944.

[ece370655-bib-0029] Dawkins, R. , and J. R. Krebs . 1978. “Animal Signals: Information or Manipulation.” Behavioural Ecology – An Evolutionary Approach 2: 282–309.

[ece370655-bib-0030] Deckel, A. W. , and E. Jevitts . 1997. “Left vs. Right‐Hemisphere Regulation of Aggressive Behaviors in *Anolis carolinensis* : Effects of Eye‐Patching and Fluoxetine Administration.” Journal of Experimental Zoology 278: 9–21.

[ece370655-bib-0031] Deng, L. , and S. Xu . 2017. “Adaptation of Human Skin Color in Various Populations.” Hereditas 155: 1. 28701907 10.1186/s41065-017-0036-2 PMC5502412

[ece370655-bib-0032] Dey, C. J. , J. Dale , and J. S. Quinn . 2014. “Manipulating the Appearance of a Badge of Status Causes Changes in True Badge Expression.” Proceedings of the Royal Society B: Biological Sciences 281: 20132680. 10.1098/rspb.2013.2680 PMC3866412 24285201

[ece370655-bib-0033] Dickerson, A. L. , K. J. Rankin , V. Cadena , J. A. Endler , and D. Stuart‐Fox . 2020. “Rapid Beard Darkening Predicts Contest Outcome, Not Copulation Success, in Bearded Dragon Lizards.” Animal Behaviour 170: 167–176.

[ece370655-bib-0034] Diep, S. K. , and D. F. Westneat . 2013. “The Integration of Function and Ontogeny in the Evolution of Status Signals.” Behaviour 150: 1015–1044.

[ece370655-bib-0035] Dijkstra, P. D. , S. M. Maguire , R. M. Harris , et al. 2017. “The Melanocortin System Regulates Body Pigmentation and Social Behaviour in a Colour Polymorphic Cichlid Fish.” Proceedings of the Royal Society B: Biological Sciences 284: 20162838. 10.1098/rspb.2016.2838 PMC5378087 28356453

[ece370655-bib-0036] Dijkstra, P. D. , S. van Dijk , T. G. G. Groothuis , M. E. R. Pierotti , and O. Seehausen . 2009. “Behavioral Dominance Between Female Color Morphs of a Lake Victoria Cichlid Fish.” Behavioral Ecology 20: 593–600.

[ece370655-bib-0037] Ducrest, A.‐L. , L. Keller , and A. Roulin . 2008. “Pleiotropy in the Melanocortin System, Coloration and Behavioural Syndromes.” Trends in Ecology & Evolution 23: 502–510. 18644658 10.1016/j.tree.2008.06.001

[ece370655-bib-0038] Duval, S. , and R. Tweedie . 2000. “Trim and Fill: A Simple Funnel‐Plot–Based Method of Testing and Adjusting for Publication Bias in Meta‐Analysis.” Biometrics 56: 455–463. 10877304 10.1111/j.0006-341x.2000.00455.x

[ece370655-bib-0039] Fisher, R. A. 1915. “Frequency Distribution of the Values of the Correlation Coefficient in Samples From an Indefinitely Large Population.” Biometrika 10: 507–521.

[ece370655-bib-0040] Foerster, K. , T. Coulson , B. C. Sheldon , J. M. Pemberton , T. H. Clutton‐Brock , and L. E. B. Kruuk . 2007. “Sexually Antagonistic Genetic Variation for Fitness in Red Deer.” Nature 447: 1107–1110. 17597758 10.1038/nature05912

[ece370655-bib-0041] Franklin, A. M. , C. M. Donatelli , C. R. Culligan , and E. D. Tytell . 2019. “Meral‐Spot Reflectance Signals Weapon Performance in the Mantis Shrimp *Neogonodactylus oerstedii* (Stomatopoda).” Biological Bulletin 236: 43–54. 30707606 10.1086/700836

[ece370655-bib-0042] Ginzburg, L. R. , and C. X. J. Jensen . 2004. “Rules of Thumb for Judging Ecological Theories.” Trends in Ecology & Evolution 19: 121–126. 16701242 10.1016/j.tree.2003.11.004

[ece370655-bib-0043] Gurevitch, J. , and L. V. Hedges . 1999. “Statistical Issues in Ecological Meta‐Analyses.” Ecology 80: 1142–1149.

[ece370655-bib-0044] Hadfield, J. D. 2010. “MCMC Methods for Multi‐Response Generalized Linear Mixed Models: The MCMCglmm R Package.” Journal of Statistical Software 33: 1–22. 20808728 PMC2929880

[ece370655-bib-0045] Hedges, S. B. , J. Dudley , and S. Kumar . 2006. “TimeTree: A Public Knowledge‐Base of Divergence Times Among Organisms.” Bioinformatics 22: 2971–2972. 17021158 10.1093/bioinformatics/btl505

[ece370655-bib-0046] Hedrick, P. W. , E. M. Tuttle , and R. A. Gonser . 2018. “Negative‐Assortative Mating in the White‐Throated Sparrow.” Journal of Heredity 109: 223–231. 29040605 10.1093/jhered/esx086 PMC6307691

[ece370655-bib-0047] Hooper, R. E. , S. J. Plaistow , and Y. Tsubaki . 2006. “Signal Function of Wing Colour in a Polymorphic Damselfly, Mnais Costalis Selys (Zygoptera: Calopterygidae).” Odonatologica 35: 15–22.

[ece370655-bib-0048] Horth, L. 2003. “Melanic Body Colour and Aggressive Mating Behaviour Are Correlated Traits in Male Mosquitofish ( *Gambusia holbrooki* ).” Proceedings of the Royal Society of London ‐ Series B: Biological Sciences 270: 1033–1040. 10.1098/rspb.2003.2348 PMC1691335 12803892

[ece370655-bib-0049] Horton, B. M. , M. E. Hauber , and D. L. Maney . 2012. “Morph Matters: Aggression Bias in a Polymorphic Sparrow.” PLoS One 7: e48705. 23119092 10.1371/journal.pone.0048705 PMC3485354

[ece370655-bib-0050] Horton, B. M. , I. T. Moore , and D. L. Maney . 2014. “New Insights Into the Hormonal and Behavioural Correlates of Polymorphism in White‐Throated Sparrows, *Zonotrichia albicollis* .” Animal Behaviour 93: 207–219. 25045171 10.1016/j.anbehav.2014.04.015 PMC4099966

[ece370655-bib-0051] Houle, D. 1991. “Genetic Covariance of Fitness Correlates: What Genetic Correlations Are Made of and Why It Matters.” Evolution 45: 630–648. 28568816 10.1111/j.1558-5646.1991.tb04334.x

[ece370655-bib-0052] Huhta, E. , and R. V. Alatalo . 1993. “Plumage Colour and Male‐Male Interactions in the Pied Flycatcher.” Animal Behaviour 45: 511–518.

[ece370655-bib-0053] Jacobs, P. , and W. Viechtbauer . 2017. “Estimation of the Biserial Correlation and Its Sampling Variance for Use in Meta‐Analysis.” Research Synthesis Methods 8: 161–180. 27631635 10.1002/jrsm.1218

[ece370655-bib-0054] Jawor, J. M. , N. Gray , S. M. Beall , and R. Breitwisch . 2004. “Multiple Ornaments Correlate With Aspects of Condition and Behaviour in Female Northern Cardinals, *Cardinalis cardinalis* .” Animal Behaviour 67: 875–882.

[ece370655-bib-0055] Johnstone, R. A. , and K. Norris . 1993. “Badges of Status and the Cost of Aggression.” Behavioral Ecology and Sociobiology 32: 127–134.

[ece370655-bib-0056] Karlsson, A.‐C. , P. Mormede , S. Kerje , and P. Jensen . 2011. “Genotype on the Pigmentation Regulating PMEL17 Gene Affects Behavior in Chickens Raised Without Physical Contact With Conspecifics.” Behavior Genetics 41: 312–322. 20623330 10.1007/s10519-010-9379-4

[ece370655-bib-0057] Kenyon, H. L. , and P. R. Martin . 2023. “Color as an Interspecific Badge of Status: A Comparative Test.” American Naturalist 202: 433–447. 10.1086/725916 37792917

[ece370655-bib-0058] Kim, Y. K. , S. S. Lee , S. I. Oh , et al. 2010. “Behavioural Reactivity of the Korean Native Jindo Dog Varies With Coat Colour.” Behavioural Processes 84: 568–572. 20176091 10.1016/j.beproc.2010.02.012

[ece370655-bib-0059] Kingma, S. A. , I. Szentirmai , T. Székely , et al. 2008. “Sexual Selection and the Function of a Melanin‐Based Plumage Ornament in Polygamous Penduline Tits *Remiz pendulinus* .” Behavioral Ecology and Sociobiology 62: 1277–1288.

[ece370655-bib-0060] Knapton, R. W. , and J. B. Falls . 1983. “Differences in Parental Contribution Among Pair Types in the Polymorphic White‐Throated Sparrow.” Canadian Journal of Zoology 61: 1288–1292.

[ece370655-bib-0061] Korsten, P. , T. H. Dijkstra , and J. Komdeur . 2007. “Is UV Signalling Involved in Male‐Male Territorial Conflict in the Blue Tit ( *Cyanistes caeruleus* )? A New Experimental Approach.” Behaviour 144: 447–470.

[ece370655-bib-0062] Korzan, W. J. , R. R. Robison , S. Zhao , and R. D. Fernald . 2008. “Color Change as a Potential Behavioral Strategy.” Hormones and Behavior 54: 463–470. 18586245 10.1016/j.yhbeh.2008.05.006 PMC3019090

[ece370655-bib-0063] Kraft, B. , V. A. Lemakos , J. Travis , and K. A. Hughes . 2018. “Pervasive Indirect Genetic Effects on Behavioral Development in Polymorphic Eastern Mosquitofish.” Behavioral Ecology 29: 289–300.

[ece370655-bib-0064] Kumar, S. , G. Stecher , M. Suleski , and S. B. Hedges . 2017. “TimeTree: A Resource for Timelines, Timetrees, and Divergence Times.” Molecular Biology and Evolution 34: 1812–1819. 28387841 10.1093/molbev/msx116

[ece370655-bib-0065] Küpper, C. , M. Stocks , J. E. Risse , et al. 2016. “A Supergene Determines Highly Divergent Male Reproductive Morphs in the Ruff.” Nature Genetics 48: 79–83. 26569125 10.1038/ng.3443 PMC5218575

[ece370655-bib-0066] Lajeunesse, M. J. 2009. “Meta‐Analysis and the Comparative Phylogenetic Method.” American Naturalist 174: 369–381. 10.1086/603628 19637963

[ece370655-bib-0067] Lamichhaney, S. , G. Fan , F. Widemo , et al. 2016. “Structural Genomic Changes Underlie Alternative Reproductive Strategies in the Ruff ( *Philomachus pugnax* ).” Nature Genetics 48: 84–88. 26569123 10.1038/ng.3430

[ece370655-bib-0068] Lank, D. B. , C. M. Smith , O. Hanotte , T. Burke , and F. Cooke . 1995. “Genetic Polymorphism for Alternative Mating Behaviour in Lekking Male Ruff *Philomachus pugnax* .” Nature 378: 59–62.

[ece370655-bib-0069] Lehtonen, T. K. 2014. “Colour Biases in Territorial Aggression in a Neotropical Cichlid Fish.” Oecologia 175: 85–93. 24414236 10.1007/s00442-013-2879-1

[ece370655-bib-0070] Leitão, A. V. , A. C. Ferreira , C. Funghi , S. Trigo , and P. G. Mota . 2015. “Evidence for Multiple Functions in a Sexually Selected Ornament.” Animal Behaviour 110: 155–161.

[ece370655-bib-0071] Leitão, A. V. , M. L. Hall , K. Delhey , and R. A. Mulder . 2019. “Female and Male Plumage Colour Signals Aggression in a Dichromatic Tropical Songbird.” Animal Behaviour 150: 285–301.

[ece370655-bib-0072] Lenth, R. , H. Singmann , J. Love , P. Buerkner , and M. Herve . 2021. “Estimated Marginal Means, Aka Least‐Squares Means. R package version 1.7. 2.” https://cran.r‐project.org/web/packages/emmeans/index.html2022.

[ece370655-bib-0073] Lipsey, M. W. , and D. B. Wilson . 2001. Practical Meta‐Analysis. Thousand Oaks, CA: Sage Publications, Inc.

[ece370655-bib-0074] Lowther, J. K. 1961. “Polymorphism in the White‐Throated Sparrow, *Zonotrichia albicollis* (Gmelin).” Canadian Journal of Zoology 39: 281–292.

[ece370655-bib-0075] Mafli, A. , K. Wakamatsu , and A. Roulin . 2011. “Melanin‐Based Coloration Predicts Aggressiveness and Boldness in Captive Eastern Hermann's Tortoises.” Animal Behaviour 81: 859–863.

[ece370655-bib-0076] Martin, F. D. , and M. F. Hengstebeck . 1981. “Eye Colour and Aggression in Juvenile Guppies, *Poecilia reticulata* Peters (Pisces: Poeciliidae).” Animal Behaviour 29: 325–331.

[ece370655-bib-0077] Massey, J. H. , and P. J. Wittkopp . 2016. “The Genetic Basis of Pigmentation Differences Within and Between Drosophila Species.” Current Topics in Developmental Biology 119: 27–61. 27282023 10.1016/bs.ctdb.2016.03.004 PMC5002358

[ece370655-bib-0078] McAdam, A. G. , S. Boutin , A. K. Sykes , and M. M. Humphries . 2007. “Life Histories of Female Red Squirrels and Their Contributions to Population Growth and Lifetime Fitness.” Écoscience 14: 362–369.

[ece370655-bib-0079] McDonald, D. B. , R. P. Clay , R. T. Brumfield , and M. J. Braun . 2001. “Sexual Selection On Plumage And Behavior In An Avian Hybrid Zone: Experimental Tests Of Male‐Male Interactions.” Evolution 55: 1443–1451. 11525466 10.1111/j.0014-3820.2001.tb00664.x

[ece370655-bib-0080] McElroy, E. J. , C. Marien , J. J. Meyers , and D. J. Irschick . 2007. “Do Displays Send Information About Ornament Structure and Male Quality in the Ornate Tree Lizard, *Urosaurus ornatus* ?” Ethology 113: 1113–1122.

[ece370655-bib-0081] McGraw, K. J. , J. Dale , and E. A. Mackillop . 2003. “Social Environment During Molt and the Expression of Melanin‐Based Plumage Pigmentation in Male House Sparrows ( *Passer domesticus* ).” Behavioral Ecology and Sociobiology 53: 116–122.

[ece370655-bib-0082] McGraw, K. J. , E. A. Mackillop , J. Dale , and M. E. Hauber . 2002. “Different Colors Reveal Different Information: How Nutritional Stress Affects the Expression of Melanin‐ and Structurally Based Ornamental Plumage.” Journal of Experimental Biology 205: 3747–3755. 12409501 10.1242/jeb.205.23.3747

[ece370655-bib-0083] McGraw, K. J. , W. Medina‐Jerez , and H. Adams . 2007. “Carotenoid‐Based Plumage Coloration and Aggression During Molt in Male House Finches.” Behaviour 144: 165–178.

[ece370655-bib-0084] Moore, M. P. , and R. A. Martin . 2016. “Intrasexual Selection Favours an Immune‐Correlated Colour Ornament in a Dragonfly.” Journal of Evolutionary Biology 29: 2256–2265. 27467980 10.1111/jeb.12953

[ece370655-bib-0085] Nakagawa, S. , N. Ockendon , D. O. Gillespie , B. J. Hatchwell , and T. Burke . 2007. “Assessing the Function of House Sparrows Bib Size Using a Flexible Meta‐Analysis Method.” Behavioral Ecology 18: 831.

[ece370655-bib-0086] Nakagawa, S. , and E. S. A. Santos . 2012. “Methodological Issues and Advances in Biological Meta‐Analysis.” Evolutionary Ecology 26: 1253–1274.

[ece370655-bib-0087] Naretto, S. , and M. Chiaraviglio . 2023. “Hoisting the White Flag of Surrender? Color Change in Agonistic Encounters Between Achala Copper Lizard Males ( *Pristidactylus achalensis* ).” Behavioral Ecology and Sociobiology 77: 116.

[ece370655-bib-0088] Nie, C. , L. Ban , Z. Ning , and L. Qu . 2019. “Feather Colour Affects the Aggressive Behaviour of Chickens With the Same Genotype on the Dominant White (I) Locus.” PLoS One 14: e0215921. 31048862 10.1371/journal.pone.0215921 PMC6497237

[ece370655-bib-0089] O'Connor, E. A. , J. E. Saunders , H. Grist , M. A. McLeman , C. M. Wathes , and S. M. Abeyesinghe . 2011. “The Relationship Between the Comb and Social Behaviour in Laying Hens.” Applied Animal Behaviour Science 135: 293–299.

[ece370655-bib-0090] O'Connor, K. I. , N. B. Metcalfe , and A. C. Taylor . 2000. “The Effects of Prior Residence on Behavior and Growth Rates in Juvenile Atlantic Salmon ( *Salmo salar* ).” Behavioral Ecology 11: 13–18.

[ece370655-bib-0091] O'Dea, R. E. , M. Lagisz , M. D. Jennions , et al. 2021. “Preferred Reporting Items for Systematic Reviews and Meta‐Analyses in Ecology and Evolutionary Biology: A PRISMA Extension.” Biological Reviews 96: 1695–1722. 33960637 10.1111/brv.12721 PMC8518748

[ece370655-bib-0092] Omar, D.‐C. 2024. “Intra‐Sexual Selection in a North American Annual Killifish: Does the Color‐Polymorphism Matter?” Acta Ethologica 27: 153–165.

[ece370655-bib-0093] Paradis, E. , J. Claude , and K. Strimmer . 2004. “APE: Analyses of Phylogenetics and Evolution in R Language.” Bioinformatics 20: 289–290. 14734327 10.1093/bioinformatics/btg412

[ece370655-bib-0094] Pärt, T. , and A. Qvarnström . 1997. “Badge Size in Collared Flycatchers Predicts Outcome of Male Competition Over Territories.” Animal Behaviour 54: 893–899. 9344442 10.1006/anbe.1997.0514

[ece370655-bib-0095] Petrocelli, I. , G. Ricciardi , A. Rodrigues de Souza , A. Ermanni , A. Ninu , and S. Turillazzi . 2015. “Visual Signals of Individual Quality in a European Solitary Founding Paper Wasp.” Ethology 121: 300–307.

[ece370655-bib-0096] Podberscek, A. L. , and J. A. Serpell . 1996. “The English Cocker Spaniel: Preliminary Findings on Aggressive Behaviour.” Applied Animal Behaviour Science 47: 75–89.

[ece370655-bib-0097] Pryke, S. R. , and S. C. Griffith . 2009. “Socially Mediated Trade‐Offs Between Aggression and Parental Effort in Competing Color Morphs.” American Naturalist 174: 455–464. 10.1086/605376 19689213

[ece370655-bib-0098] Pryke, S. R. , M. J. Lawes , and S. Andersson . 2001. “Agonistic Carotenoid Signalling in Male Red‐Collared Widowbirds: Aggression Related to the Colour Signal of Both the Territory Owner and Model Intruder.” Animal Behaviour 62: 695–704.

[ece370655-bib-0099] Pustejovsky, J. E. 2014. “Converting From d to r to z When the Design Uses Extreme Groups, Dichotomization, or Experimental Control.” Psychological Methods 19: 92–112. 24079923 10.1037/a0033788

[ece370655-bib-0100] R Core Team . 2023. R: A Language and Environment for Statistical Computing. Vienna, Austria: R Foundation for Statistical Computing.

[ece370655-bib-0101] Reissmann, M. , and A. Ludwig . 2013. “Pleiotropic Effects of Coat Colour‐Associated Mutations in Humans, Mice and Other Mammals.” Seminars in Cell & Developmental Biology 24: 576–586. 23583561 10.1016/j.semcdb.2013.03.014

[ece370655-bib-0102] Reiter, M. K. , C. D. Anthony , and C.‐A. M. Hickerson . 2014. “Territorial Behavior and Ecological Divergence in a Polymorphic Salamander.” Copeia 2014: 481–488.

[ece370655-bib-0103] Renn, S. C. P. , E. J. Fraser , N. Aubin‐Horth , B. C. Trainor , and H. A. Hofmann . 2012. “Females of an African Cichlid Fish Display Male‐Typical Social Dominance Behavior and Elevated Androgens in the Absence of Males.” Hormones and Behavior 61: 496–503. 22285646 10.1016/j.yhbeh.2012.01.006 PMC3319202

[ece370655-bib-0104] Revell, L. J. 2012. “Phytools: An R Package for Phylogenetic Comparative Biology (And Other Things).” Methods in Ecology and Evolution 3: 217–223.

[ece370655-bib-0105] Robertson, J. M. , and E. B. Rosenblum . 2010. “Male Territoriality and ‘Sex Confusion’ in Recently Adapted Lizards at White Sands.” Journal of Evolutionary Biology 23: 1928–1936. 20695966 10.1111/j.1420-9101.2010.02063.x

[ece370655-bib-0106] Rodriguez‐Barreto, D. , O. Rey , T. M. Uren‐Webster , G. Castaldo , S. Consuegra , and C. Garcia de Leaniz . 2019. “Transcriptomic Response to Aquaculture Intensification in *Nile tilapia* .” Evolutionary Applications 12: 1757–1771. 31548855 10.1111/eva.12830 PMC6752142

[ece370655-bib-0107] Rohwer, S. 1975. “The Social Significance of Avian Winter Plumage Variability.” Evolution 29: 593–610. 28563094 10.1111/j.1558-5646.1975.tb00853.x

[ece370655-bib-0108] Rose, P. , and L. Soole . 2020. “What Influences Aggression and Foraging Activity in Social Birds? Measuring Individual, Group and Environmental Characteristics.” Ethology 126: 900–913.

[ece370655-bib-0109] Rosenthal, R. 1979. “The File Drawer Problem and Tolerance for Null Results.” Psychological Bulletin 86: 638–641.

[ece370655-bib-0110] Roulin, A. 2016. “Condition‐Dependence, Pleiotropy and the Handicap Principle of Sexual Selection in Melanin‐Based Colouration.” Biological Reviews 91: 328–348. 25631160 10.1111/brv.12171

[ece370655-bib-0111] Roulin, A. , and A.‐L. Ducrest . 2011. “Association Between Melanism, Physiology and Behaviour: A Role for the Melanocortin System.” European Journal of Pharmacology 660: 226–233. 21300052 10.1016/j.ejphar.2011.01.036

[ece370655-bib-0112] Roulin, A. , J. Gasparini , P. Bize , M. Ritschard , and H. Richner . 2008. “Melanin‐Based Colorations Signal Strategies to Cope With Poor and Rich Environments.” Behavioral Ecology and Sociobiology 62: 507–519.

[ece370655-bib-0113] Rudh, A. , M. F. Breed , and A. Qvarnström . 2013. “Does Aggression and Explorative Behaviour Decrease With Lost Warning Coloration?” Biological Journal of the Linnean Society 108: 116–126.

[ece370655-bib-0114] Sánchez‐Tójar, A. , S. Nakagawa , M. Sánchez‐Fortún , et al. 2018. “Meta‐Analysis Challenges a Textbook Example of Status Signalling and Demonstrates Publication Bias.” eLife 7: e37385. 30420005 10.7554/eLife.37385 PMC6234027

[ece370655-bib-0115] San‐Jose, L. M. , and A. Roulin . 2018. “Toward Understanding the Repeated Occurrence of Associations Between Melanin‐Based Coloration and Multiple Phenotypes.” American Naturalist 192: 111–130. 10.1086/698010 30016163

[ece370655-bib-0116] Santos, E. S. A. , D. Scheck , and S. Nakagawa . 2011. “Dominance and Plumage Traits: Meta‐Analysis and Metaregression Analysis.” Animal Behaviour 82: 3–19.

[ece370655-bib-0117] Santostefano, F. , K. V. Fanson , J. A. Endler , and P. A. Biro . 2019. “Behavioral, Energetic, and Color Trait Integration in Male Guppies: Testing the Melanocortin Hypothesis.” Behavioral Ecology 30: 1539–1547.

[ece370655-bib-0118] Schartl, M. , L. Larue , M. Goda , M. W. Bosenberg , H. Hashimoto , and R. N. Kelsh . 2016. “What Is a Vertebrate Pigment Cell?” Pigment Cell & Melanoma Research 29: 8–14. 26247887 10.1111/pcmr.12409

[ece370655-bib-0119] Schwarzer, G. 2007. “Meta: An R Package for Meta‐Analysis.” R News 7: 40–45.

[ece370655-bib-0120] Seaver, C. M. S. , and P. L. Hurd . 2017. “Are There Consistent Behavioral Differences Between Sexes and Male Color Morphs in *Pelvicachromis pulcher* ?” Zoology 122: 115–125. 28546067 10.1016/j.zool.2017.05.002

[ece370655-bib-0121] Seddon, R. J. , and D. K. Hews . 2016. “Phenotypic Correlates of Melanization in Two *Sceloporus occidentalis* (Phrynosomatidae) Populations: Behavior, Androgens, Stress Reactivity, and Ectoparasites.” Physiology & Behavior 163: 70–80. 27137079 10.1016/j.physbeh.2016.04.039

[ece370655-bib-0122] Seddon, R. J. , and D. K. Hews . 2017. “Correlates of Melanization in Multiple High‐ and Low‐Elevation Populations of the Lizard, *Sceloporus occidentalis* : Behavior, Hormones, and Parasites.” Journal of Experimental Zoology. Part A, Ecological and Integrative Physiology 327: 481–492. 29356435 10.1002/jez.2133

[ece370655-bib-0123] Senior, A. M. , C. E. Grueber , T. Kamiya , et al. 2016. “Heterogeneity in Ecological and Evolutionary Meta‐Analyses: Its Magnitude and Implications.” Ecology 97: 3293–3299. 27912008 10.1002/ecy.1591

[ece370655-bib-0124] Siefferman, L. , and G. E. Hill . 2003. “Structural and Melanin Coloration Indicate Parental Effort and Reproductive Success in Male Eastern Bluebirds.” Behavioral Ecology 14: 855–861.

[ece370655-bib-0125] Signorell, A. , K. Aho , A. Alfons , et al. 2021. “DescTools: Tools for Descriptive Statistics.” R Package Version 0.99.44. Available online: https://cran.r‐project.org/package=DescTools.

[ece370655-bib-0126] Silver, N. C. , and W. P. Dunlap . 1987. “Averaging Correlation Coefficients: Should Fisher's z Transformation Be Used?” Journal of Applied Psychology 72: 146–148.

[ece370655-bib-0127] Sinervo, B. , and C. M. Lively . 1996. “The Rock–Paper–Scissors Game and the Evolution of Alternative Male Strategies.” Nature 380: 240–243.

[ece370655-bib-0128] Smith, M. R. , and H. Wickham . 2019. “TreeTools: Create, Modify and Analyse Phylogenetic Trees.” Comprehensive R Archive Network. R package version 1.12.0. 10.5281/zenodo.3522725.

[ece370655-bib-0129] Spiegelhalter, D. J. , N. G. Best , B. P. Carlin , and A. Van Der Linde . 2002. “Bayesian Measures of Model Complexity and Fit.” Journal of the Royal Statistical Society. Series B, Statistical Methodology 64: 583–639.

[ece370655-bib-0130] Stapley, J. 2006. “Individual Variation in Preferred Body Temperature Covaries With Social Behaviours and Colour in Male Lizards.” Journal of Thermal Biology 31: 362–369.

[ece370655-bib-0131] Suter, H. C. , and F. A. Huntingford . 2002. “Eye Colour in Juvenile Atlantic Salmon: Effects of Social Status, Aggression and Foraging Success.” Journal of Fish Biology 61: 606–614.

[ece370655-bib-0132] Taylor, J. N. , and M. S. Lattanzio . 2016. “Boldness, Dominance, and Territoriality in the Color Polymorphic Tree Lizard, *Urosaurus ornatus* .” Ethology 122: 892–901.

[ece370655-bib-0133] Tibbetts, E. A. , and J. Dale . 2004. “A Socially Enforced Signal of Quality in a Paper Wasp.” Nature 432: 218–222. 15538369 10.1038/nature02949

[ece370655-bib-0134] Tinghitella, R. M. , W. R. Lehto , and V. F. Lierheimer . 2018. “Color and Behavior Differently Predict Competitive Outcomes for Divergent Stickleback Color Morphs.” Current Zoology 64: 115–123. 29492044 10.1093/cz/zox070 PMC5809037

[ece370655-bib-0135] Tinghitella, R. M. , W. R. Lehto , and R. Minter . 2015. “The Evolutionary Loss of a Badge of Status Alters Male Competition in Three‐Spine Stickleback.” Behavioral Ecology 26: 609–616.

[ece370655-bib-0136] Titone, V. , F. Marsiglia , M. Mangiacotti , R. Sacchi , S. Scali , and M. a. L. Zuffi . 2018. “Better to Be Resident, Larger or Coloured? Experimental Analysis on Intraspecific Aggression in the Ruin Lizard.” Journal of Zoology 304: 260–267.

[ece370655-bib-0137] Tuttle, E. M. 2003. “Alternative Reproductive Strategies in the White‐Throated Sparrow: Behavioral and Genetic Evidence.” Behavioral Ecology 14: 425–432.

[ece370655-bib-0138] Tuttle, E. M. , A. O. Bergland , M. L. Korody , et al. 2016. “Divergence and Functional Degradation of a Sex Chromosome‐Like Supergene.” Current Biology 26: 344–350. 26804558 10.1016/j.cub.2015.11.069 PMC4747794

[ece370655-bib-0139] Vercken, E. , and J. Clobert . 2008. “The Role of Colour Polymorphism in Social Encounters Among Female Common Lizards.” Herpetological Journal 18: 223–230.

[ece370655-bib-0140] Walsh, B. , and M. W. Blows . 2009. “Abundant Genetic Variation + Strong Selection = Multivariate Genetic Constraints: A Geometric View of Adaptation.” Annual Review of Ecology, Evolution, and Systematics 40: 41–59.

[ece370655-bib-0141] Wellenreuther, M. , E. I. Svensson , and B. Hansson . 2014. “Sexual Selection and Genetic Colour Polymorphisms in Animals.” Molecular Ecology 23: 5398–5414. 25251393 10.1111/mec.12935

[ece370655-bib-0142] Williams, L. J. , A. J. King , and C. Mettke‐Hofmann . 2012. “Colourful Characters: Head Colour Reflects Personality in a Social Bird, the Gouldian Finch, *Erythrura gouldiae* .” Animal Behaviour 84: 159–165.

[ece370655-bib-0143] Wittkopp, P. J. , and P. Beldade . 2009. “Development and Evolution of Insect Pigmentation: Genetic Mechanisms and the Potential Consequences of Pleiotropy.” Seminars in Cell & Developmental Biology 20: 65–71. 18977308 10.1016/j.semcdb.2008.10.002

[ece370655-bib-0144] Yang, Y. , M. B. Dugas , H. J. Sudekum , S. N. Murphy , and C. L. Richards‐Zawacki . 2018. “Male–Male Aggression Is Unlikely to Stabilize a Poison Frog Polymorphism.” Journal of Evolutionary Biology 31: 457–468. 29345026 10.1111/jeb.13243

[ece370655-bib-0145] Yewers, M. S. C. , S. Pryke , and D. Stuart‐Fox . 2016. “Behavioural Differences Across Contexts May Indicate Morph‐Specific Strategies in the Lizard *Ctenophorus decresii* .” Animal Behaviour 111: 329–339.

[ece370655-bib-0146] Yong, L. , B. Lee , and J. S. McKinnon . 2018. “Variation in Female Aggression in 2 Three‐Spined Stickleback Populations With Female Throat and Spine Coloration.” Current Zoology 64: 345–350. 30402077 10.1093/cz/zoy020 PMC6007237

[ece370655-bib-0147] Yong, L. , B. E. Woodall , M. E. R. Pierotti , and J. S. McKinnon . 2015. “Intrasexual Competition and Throat Color Evolution in Female Three‐Spined Sticklebacks.” Behavioral Ecology 26: 1030–1038.

[ece370655-bib-0148] Zinzow‐Kramer, W. M. , B. M. Horton , C. D. McKee , et al. 2015. “Genes Located in a Chromosomal Inversion Are Correlated With Territorial Song in White‐Throated Sparrows.” Genes, Brain, and Behavior 14: 641–654. 26463687 10.1111/gbb.12252 PMC4785874

