# Supplementary material for: Assessing the Association Between Animal Color and Behavior: A Meta‐Analysis of Experimental Studies
Source: Ecol Evol. 2024 Dec 4;14(12):e70655. doi: 10.1002/ece3.70655 (PMC11617328; doi:10.1002/ece3.70655)
Supplement: Supplementary file 1 — Figure S1. Phylogeny for papers examined. The phylogeny is an ultrametric tree that was fully resolved to the species level, which we obtained from TimeTree.org. [file ECE3-14-e70655-s005.docx]

Supplemental Figures:


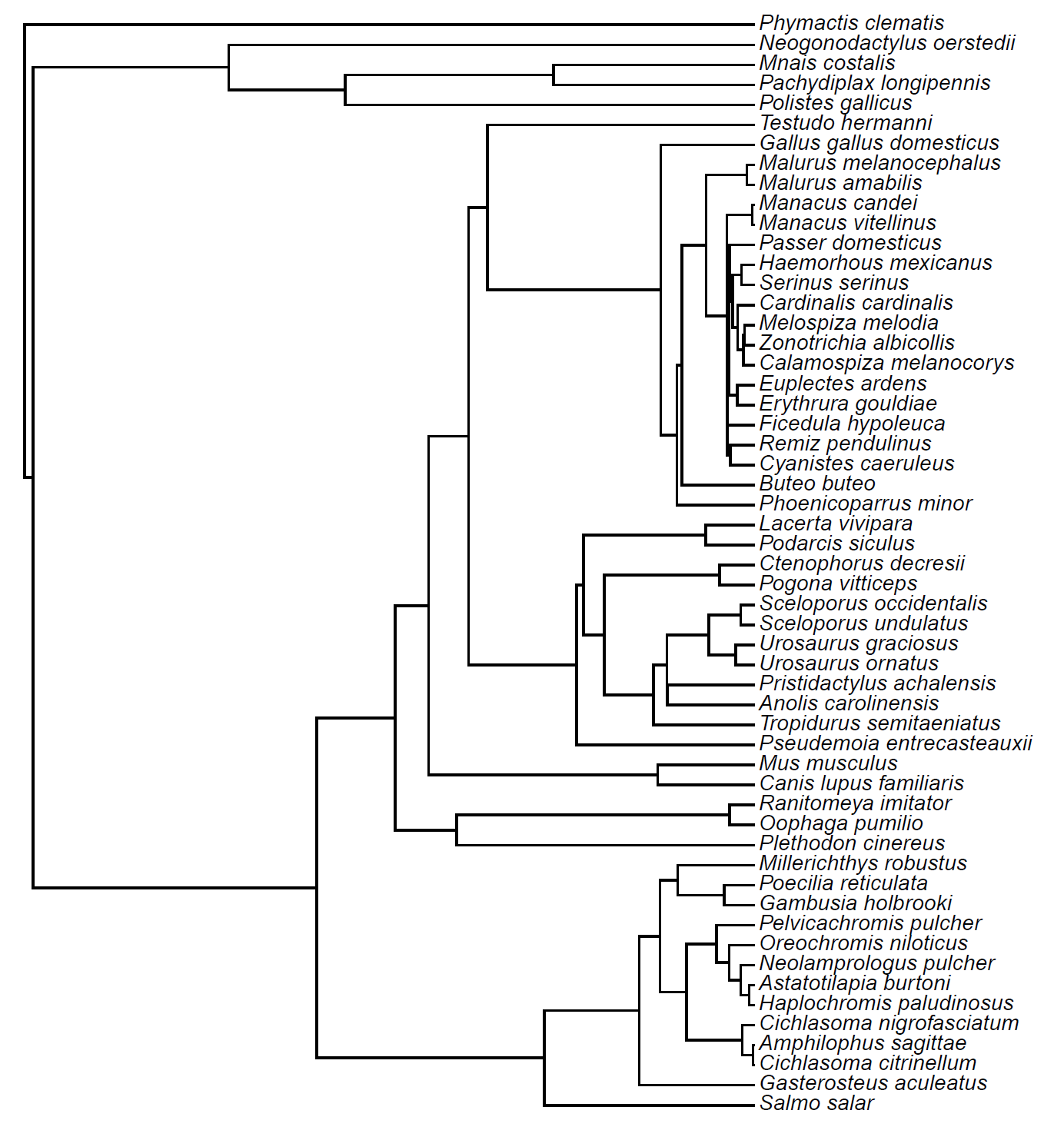


Supplemental Figure S1: Phylogeny for Papers Examined. The phylogeny is an ultrametric tree that was fully resolved to the species level, which we obtained from TimeTree.org.
