# Supplementary material for: Assessing the Association Between Animal Color and Behavior: A Meta‐Analysis of Experimental Studies
Source: Ecol Evol. 2024 Dec 4;14(12):e70655. doi: 10.1002/ece3.70655 (PMC11617328; doi:10.1002/ece3.70655)
Supplement: Supplementary file 2 — Table S1. Body color classification justification. Every species included in the meta‐analysis is listed, along with categories of the colors used by the study authors (Columns 1–3). Justification for the classification of the colors used in the meta‐analysis is in Columns 6–7. [file ECE3-14-e70655-s004.docx]

Supplemental Table S1: Body Color Classification Justification. Every species included in the meta-analysis is listed, along with categories of the colors used by the study authors (columns 1-3). Justification for the classification of the colors used in the meta-analysis are in columns 6-7.

| **Species** | **Light Color** | **Dark Color** | **Plastic** | **Breeding** | **Classification** | **Explanation** |
| --- | --- | --- | --- | --- | --- | --- |
| *Amphilophus sagittae* | Gold | Black/dark | No | Yes | Melanin  (eumelanin) | Melanophore death = gold and black is due to melanophore presence (Henning et al. 2010, 2013) |
| *Anolis carolinensis* | Light green | Dark green or Brown | Yes | No | Melanin (eumelanin) | Melanophore location under the xanthophores. Below = green and above = brown (Taylor and Hadley 1970) |
| *Astatotilapia burtoni* | Blue | Yellow | Yes | No | Melanocortin (eumelanin) | Exogenous α-melanocyte-stimulating hormone (α-MSH) increases yellowness of the body and dispersal of xanthophore pigments in both morphs (Dijkstra et al. 2017) |
| *Astatotilapia burtoni* | No black | Black | No | No | Melanocortin (eumelanin) | Eumelanin = black bars on males (Li et al. 2021) |
| *Buteo Buteo* | Light white | Dark/brown | Yes | No | Melanocortin (eumelanin) | Eumelanic morphs (Chakarov et al. 2008) |
| *Calamospiza melanocorys* | Light black | Dark black | No | Yes | Melanocortin (eumelanin) | The black breeding plumage of males results from melanin deposition during feather growth (Chaine n.d.). |
| *Canis lupus familiaris* | White | Fawn | No | No | Melanocortin (pheomelanin) | Fawn is based on the agouti gene and is based on pheomelanin (Schmutz and Berryere 2007) |
| *Canis lupus familiaris* | Black | Red/Gold | No | No | Melanocortin (pheomelanin) | E gene = pheomelanin when red (Schmutz and Berryere 2007) |
| *Cardinalis cardinalis* | Light black | Dark black | No | No | Melanocortin (eumelanin) | (Jawor and Breitwisch 2003) |
| *Cichlacoma citrinellum* | Grey | Golden | Yes | No | Carotenoid | No melanophores in gold only carotenoids (Webber et al. 1973) |
| *Cichlasoma nigrofasciatum* | Dull orange | Dark orange | Yes | No | Carotenoid | Carotenoids produce brighter orange (Barlow 1976) |
| *Ctenophorus decresii* | Grey/Yellow | Orange | Yes | No | Carotenoid | Yellow is generated primarily by carotenoids and orange is generated by the combination of carotenoids and drosopterin (pteridine) (Rankin et al. 2016) |
| *Cyanistes caeruleus* | Less blue | Bluer | Yes | No | Structural | Crown of the blue tit is structural colors and changes over seasons (Delhey et al. 2010) |
| *Erythrura gouldiae* | Red | Black | Yes | No | Melanocortin (eumelanin) | Recessive alleles produce eumelanin (black melanin), which masks the effects of the carotenoids responsible for red/yellow headedness and produces the black-headed morph (Brush and Seifried 1968) |
| *Euplectes ardens* | Yellow/Orange | Red | Yes | No | Carotenoid | (Prager and Andersson 2010) |
| *Ficedula hypoleuca* | Dull brown | Dark brown | Yes | No | Melanocortin (eumelanin) | More eumelanin pigment (responsible for darker colors) than pheomelanin (which is involved in browns) (Potti et al. 2014). |
| *Gallus gallus domesticus* | White | Wild type (red) | No | No | Melanocortin (eumelanin) | Dark red pigment = eumelanin (Nätt et al. 2007) |
| *Gambusia holbrooki* | Silver | Black | No | No | Melanocortin (eumelanin) | (Angus 1989) or temperature dependent (Horth 2003) |
| *Gasterosteus aculeatus* | Dull or no red | Bright red | Yes | Yes | Carotenoid | (Brush and Reisman 1965) |
| *Gasterosteus aculeatus* | Red | Black | No | No | Melanocortin (eumelanin) | (Lewandowski and Boughman 2008) |
| *Haemorhous mexicanus* | Light red | Dark red | No | No | Carotenoid | (Lendvai et al. 2013) |
| *Haplochromis paludinosus* | Red | Blue | No | No | Unknown | Not described |
| *Haplochromis paludinosus* | Plain (orange) | Black | No | No | Melanocortin (eumelanin) | (Dijkstra et al. 2009) |
| *Lacerta vivipara* | Orange | Yellow | Yes | No | Carotenoid | Carotenoids reflect genetically based color morphs, but iridophores lead to plasticity in color (San-Jose et al. 2013) |
| *Luscinia cyanura* | Brown | Blue | No | No | Structural | (Morimoto et al. 2006) |
| *Malurus amabilis* | Grey/less blue | Blue | No | Yes | Structural | (Fan et al. 2019) |
| *Malurus melanocephalus* | Brown | Red/Black | No | Yes | Carotenoid | (Khalil et al. 2020) |
| *Manacus candei* | White | Golden | No | Yes | Carotenoid | (Bennett et al. 2021) |
| *Manacus vitellinus* | White/lemon | Golden | No | Yes | Carotenoid | (Bennett et al. 2021) |
| *Melospiza melodia* | Light brown | Dark brown | No | Yes | Melanocortin (eumelanin) | Darker = eumelanin and brown = pheomelanin (Beck et al. 2018) |
| *Millerichthys robustus* | Yellow | Red | No | No | Unknown | (Omar 2024) |
| *Mnais costalis* | Clear | Orange | Yes | No | Carotenoid | (Maoka et al. 2020) |
| *Mus musculus* | Non-agouti | Agouti | No | No | Melanocortin (pheomelanin) | (Lamoreux et al. 2001) |
| *Neogonodactylus oerstedii* | Light meral spots | Dark meral spots | No | No | Carotenoid | Carotenoproteins create the spots (Franklin et al. 2019) |
| *Neolamprologus pulcher* | No black stripes | Black stripes | No | No | Melanocortin (eumelanin) | (Balzarini et al. 2017) |
| *Oophaga pumilio* | Light red | Dark red | No | No | Carotenoid | (Rodríguez et al. 2020) |
| *Oophaga pumilio* | Green | Red | No | No | Carotenoid | (Rodríguez et al. 2020) |
| *Oophaga pumilio* | Red | Blue | No | No | Carotenoid | Carotenoid leads to blue skin (Rodríguez et al. 2020) |
| *Oreochromis niloticus* | Light (yellow) | Darker (red) | Yes | No | Carotenoid | Diet carotenoids lead to color differences (Wang et al. 2021) |
| *Pachydiplax longipennis* | Less black | Blacker | No | No | Melanocortin (eumelanin) | Most likely eumelanin (Moore and Martin 2016) |
| *Passer domesticus* | Less black | Blacker | No | No | Melanocortin (eumelanin) | Black eumelanin based feathers (Rojas Mora et al. 2016) |
| *Pelvicachromis pulcher* | Yellow | Red | No | No | Unknown | Not described. May be carotenoids since it’s a fish. |
| *Phoenicoparrus minor* | White | Pink | Yes | No | Carotenoid | Dietary carotenoids (Fox et al. 1967) |
| *Phymactis clematis* | Green | Red | No | No | Unknown | Not described. |
| *Plethodon cinereus* | Unstriped black | Striped red | No | No | Carotenoid | Erythrophores present in red backed but not black backed forms (Bagnara and Taylor 1970) |
| *Podarcis siculus* | White | Green | No | No | Unknown | Not described. |
| *Poecilia reticulata* | Less black eye | Darker eyes | Yes | No | Unknown | Not described. |
| *Pogona vitticeps* | Lighter | Darker | Yes | No | Melanocortin (eumelanin) | (Dickerson et al. 2020) |
| *Polistes gallicus* | Less black | Blacker | No | No | Melanocortin (eumelanin) | Clypeus spots (most likely eumelanin) (Tibbetts and Dale 2004) |
| *Pristidactylus achalensis* | Lighter | Darker | Yes | Yes | Unknown | They do not know what causes the darkening/lightening of males (Naretto and Chiaraviglio 2023) |
| *Pseudemoia entrecasteauxii* | White | Orange | Yes | No | Carotenoid | Most likely carotenoids (López et al. 2009) |
| *Ranitomeya imitator* | Less red | More red | No | No | Multiple - Melanocortin (eumelanin) | All types of color are expressed to create the color and mc1r is upregulated in red/orange (Rubio et al. 2024) |
| *Ranitomeya imitator* | Intermediate | Striped | No | No | Multiple - Melanocortin (eumelanin) | See above. Same principles apply. |
| *Remiz pendulinus* | Smaller patch | Larger patch | No | No | Melanocortin (eumelanin) | (Kingma et al. 2008) |
| *Salmo salar* | Pale/grey | Dark grey | No | No | Melanocortin (eumelanin) | Darker spots due to eumelanin (Kittilsen et al. 2009) |
| *Salmo salar* | Light eyes | Dark eyes | Yes | No | Unknown | Not described. |
| *Sceloporus occidentalis* | White | Black | No | No | Melanocortin (eumelanin) | (Seddon and Hews 2016) |
| *Sceloporus undulatus* | Light orange | Dark orange | No | No | Pteridine | Not carotenoids but instead pteridines lead to orange coloration (Morrison et al. 1995) |
| *Serinus serinus* | Less yellow | More yellow | Yes | No | Carotenoid | Carotenoids lead to yellow coloration (Stradi et al. 1995) |
| *Testudo hermanni* | Light brown | Dark brown | Yes | No | Melanocortin (pheomelanin) | Turtles with brown shells produce pheomelanin (Roulin et al. 2013) |
| *Tropidurus semitaeniatus* | Yellow | Black | No | Yes | Melanocortin (eumelanin) | Do not say if it is eumelanin or not but is most likely eumelanin because of other lizard species (Bruinjé et al. 2019) |
| *Urosaurus graciosus* | Yellow | Orange | No | No | Carotenoid | Related species orange morph due to carotenoids (Haisten et al. 2015) |
| *Urosaurus ornatus* | Yellow/orange | Blue | No | No | Structural | Related species blue morph due to iridophores (Haisten et al. 2015) |
| *Zonotrichia albicollis* | White | Tan | No | No | Melanocortin (pheomelanin) | Reddish brown feathers = pheomelanin (Morrow and Morrow 2020) |

References

Angus, R. A. 1989. Inheritance of Melanistic Pigmentation in the Eastern Mosquitofish. J. Hered. 80:387–392.

Bagnara, J. T., and J. D. Taylor. 1970. Differences in pigment-containing organelles between color forms of the red-backed salamander, Plethodon cinereus. Z. Für Zellforsch. Mikrosk. Anat. 106:412–417.

Balzarini, V., M. Taborsky, F. Villa, and J. G. Frommen. 2017. Computer animations of color markings reveal the function of visual threat signals in Neolamprologus pulcher. Curr. Zool. 63:45–54.

Barlow, G. W. 1976. The Midas Cichlid In Nicaragua. Investig. Ichthyofauna Nicar. Lakes 28.

Beck, M. L., S. Davies, and K. B. Sewall. 2018. Urbanization alters the relationship between coloration and territorial aggression, but not hormones, in song sparrows. Anim. Behav. 142:119–128.

Bennett, K. F. P., H. C. Lim, and M. J. Braun. 2021. Sexual Selection and Introgression in Avian Hybrid Zones: Spotlight on *Manacus*. Integr. Comp. Biol. 61:1291–1309.

Bruinjé, A. C., F. E. A. Coelho, T. M. A. Paiva, and G. C. Costa. 2019. Aggression, color signaling, and performance of the male color morphs of a Brazilian lizard (Tropidurus semitaeniatus). Behav. Ecol. Sociobiol. 73:72.

Brush, A. H., and H. M. Reisman. 1965. The carotenoid pigments in the three-spined stickleback, Gasterosteus aculeatus. Comp. Biochem. Physiol. 14:121–125.

Brush, A. H., and H. Seifried. 1968. Pigmentation and Feather Structure in Genetic Variants of the Gouldian Finch, Poephila gouldiae. The Auk 85:416–430. American Ornithological Society.

Chaine, A. S. n.d. The *evolution of multiple sexual signals in a passerine: Trait structure and selection in a dynamic world. University of California, Santa Cruz, United States -- California.

Chakarov, N., M. Boerner, and O. Krüger. 2008. Fitness in common buzzards at the cross-point of opposite melanin–parasite interactions. Funct. Ecol. 22:1062–1069.

Delhey, K., C. Burger, W. Fiedler, and A. Peters. 2010. Seasonal Changes in Colour: A Comparison of Structural, Melanin- and Carotenoid-Based Plumage Colours. PLOS ONE 5:e11582. Public Library of Science.

Dickerson, A. L., K. J. Rankin, V. Cadena, J. A. Endler, and D. Stuart-Fox. 2020. Rapid beard darkening predicts contest outcome, not copulation success, in bearded dragon lizards. Anim. Behav. 170:167–176.

Dijkstra, P. D., S. M. Maguire, R. M. Harris, A. A. Rodriguez, R. S. DeAngelis, S. A. Flores, and H. A. Hofmann. 2017. The melanocortin system regulates body pigmentation and social behaviour in a colour polymorphic cichlid fish†. Proc. R. Soc. B Biol. Sci. 284:20162838. Royal Society.

Dijkstra, P. D., S. van Dijk, T. G. G. Groothuis, M. E. R. Pierotti, and O. Seehausen. 2009. Behavioral dominance between female color morphs of a Lake Victoria cichlid fish. Behav. Ecol. 20:593–600.

Fan, M., L. D’alba, M. D. Shawkey, A. Peters, and K. Delhey. 2019. Multiple components of feather microstructure contribute to structural plumage colour diversity in fairy-wrens. Biol. J. Linn. Soc. 128:550–568.

Fox, D. L., V. E. Smith, and A. A. Wolfson. 1967. Carotenoid selectivity in blood and feathers of lesser (African), chilean and greater (European) flamingos. Comp. Biochem. Physiol. 23:225–232.

Franklin, A. M., C. M. Donatelli, C. R. Culligan, and E. D. Tytell. 2019. Meral-Spot Reflectance Signals Weapon Performance in the Mantis Shrimp Neogonodactylus oerstedii (Stomatopoda). Biol. Bull. 236:43–54. The University of Chicago Press.

Haisten, D. C., D. Paranjpe, S. Loveridge, and B. Sinervo. 2015. The Cellular Basis of Polymorphic Coloration in Common Side-Blotched Lizards, Uta stansburiana. Herpetologica 71:125–135.

Henning, F., J. C. Jones, P. Franchini, and A. Meyer. 2013. Transcriptomics of morphological color change in polychromatic Midas cichlids. BMC Genomics 14:171.

Henning, F., A. J. Renz, S. Fukamachi, and A. Meyer. 2010. Genetic, Comparative Genomic, and Expression Analyses of the Mc1r Locus in the Polychromatic Midas Cichlid Fish (Teleostei, Cichlidae Amphilophus sp.) Species Group. J. Mol. Evol. 70:405–412.

Horth, L. 2003. Melanic body colour and aggressive mating behaviour are correlated traits in male mosquitofish (Gambusia holbrooki). Proc. R. Soc. Lond. B Biol. Sci. 270:1033–1040. Royal Society.

Jawor, J. M., and R. Breitwisch. 2003. Melanin Ornaments, Honesty, and Sexual Selection. The Auk 120:249–265.

Khalil, S., J. F. Welklin, K. J. McGraw, J. Boersma, H. Schwabl, M. S. Webster, and J. Karubian. 2020. Testosterone regulates CYP2J19-linked carotenoid signal expression in male red-backed fairywrens (Malurus melanocephalus). Proc. R. Soc. B Biol. Sci. 287:20201687. Royal Society.

Kingma, S. A., I. Szentirmai, T. Székely, V. Bókony, M. Bleeker, A. Liker, and J. Komdeur. 2008. Sexual selection and the function of a melanin-based plumage ornament in polygamous penduline tits Remiz pendulinus. Behav. Ecol. Sociobiol. 62:1277–1288.

Kittilsen, S., J. Schjolden, I. Beitnes-Johansen, J. C. Shaw, T. G. Pottinger, C. Sørensen, B. O. Braastad, M. Bakken, and Ø. Øverli. 2009. Melanin-based skin spots reflect stress responsiveness in salmonid fish. Horm. Behav. 56:292–298.

Lamoreux, M. L., K. Wakamatsu, and S. Ito. 2001. Interaction of Major Coat Color Gene Functions in Mice as Studied by Chemical Analysis of Eumelanin and Pheomelanin. Pigment Cell Res. 14:23–31.

Lendvai, Á. Z., M. Giraudeau, J. Németh, V. Bakó, and K. J. McGraw. 2013. Carotenoid-based plumage coloration reflects feather corticosterone levels in male house finches (Haemorhous mexicanus). Behav. Ecol. Sociobiol. 67:1817–1824.

Lewandowski, E., and J. Boughman. 2008. Effects of genetics and light environment on colour expression in threespine sticklebacks. Biol. J. Linn. Soc. 94:663–673.

Li, C.-Y., J. R. Steighner, G. Sweatt, T. R. Thiele, and S. A. Juntti. 2021. Manipulation of the Tyrosinase gene permits improved CRISPR/Cas editing and neural imaging in cichlid fish. Sci. Rep. 11:15138. Nature Publishing Group.

López, P., M. Gabirot, and J. Martín. 2009. Immune challenge affects sexual coloration of male Iberian wall lizards. J. Exp. Zool. Part Ecol. Genet. Physiol. 311A:96–104.

Maoka, T., N. Kawase, T. Ueda, and R. Nishida. 2020. Carotenoids of dragonflies, from the perspective of comparative biochemical and chemical ecological studies. Biochem. Syst. Ecol. 89:104001.

Moore, M. P., and R. A. Martin. 2016. Intrasexual selection favours an immune-correlated colour ornament in a dragonfly. J. Evol. Biol. 29:2256–2265.

Morimoto, G., N. Yamaguchi, and K. Ueda. 2006. Plumage color as a status signal in male–male interaction in the red-flanked bushrobin, Tarsiger cyanurus. J. Ethol. 24:261–266.

Morrison, R. L., M. S. Rand, and S. K. Frost-Mason. 1995. Cellular Basis of Color Differences in Three Morphs of the Lizard Sceloporus undulatus erythrocheilus. Copeia 1995:397–408. [American Society of Ichthyologists and Herpetologists (ASIH), Allen Press].

Morrow, J., and L. Morrow. 2020. White-throated Sparrow (Zonotrichia albicollis) with Dilute Pastel Plumage. 69:8–12.

Naretto, S., and M. Chiaraviglio. 2023. Hoisting the white flag of surrender? Color change in agonistic encounters between Achala copper lizard males (Pristidactylus achalensis). Behav. Ecol. Sociobiol. 77:116.

Nätt, D., S. Kerje, L. Andersson, and P. Jensen. 2007. Plumage Color and Feather Pecking—Behavioral Differences Associated with PMEL17 Genotypes in Chicken (Gallus gallus). Behav. Genet. 37:399–407.

Omar, D.-C. 2024. Intra-sexual selection in a North American annual killifish: does the color-polymorphism matter? Acta Ethologica, doi: 10.1007/s10211-024-00444-w.

Potti, J., D. Canal, and C. Camacho. 2014. Ontogenetic variation in the plumage colour of female European Pied Flycatchers Ficedula hypoleuca. Ibis 156:879–884.

Prager, M., and S. Andersson. 2010. Convergent Evolution of Red Carotenoid Coloration in Widowbirds and Bishops (euplectes Spp.). Evolution 64:3609–3619.

Rankin, K. J., C. A. McLean, D. J. Kemp, and D. Stuart-Fox. 2016. The genetic basis of discrete and quantitative colour variation in the polymorphic lizard, Ctenophorus decresii. BMC Evol. Biol. 16:179.

Rodríguez, A., N. I. Mundy, R. Ibáñez, and H. Pröhl. 2020. Being red, blue and green: the genetic basis of coloration differences in the strawberry poison frog (Oophaga pumilio). BMC Genomics 21:301.

Rojas Mora, A., M. Meniri, G. Glauser, A. Vallat, and F. Helfenstein. 2016. Badge Size Reflects Sperm Oxidative Status within Social Groups in the House Sparrow Passer domesticus. Front. Ecol. Evol. 4.

Roulin, A., A. Mafli, and K. Wakamatsu. 2013. Reptiles Produce Pheomelanin: Evidence in the Eastern Hermann’s Tortoise (Eurotestudo boettgeri). J. Herpetol. 47:258–261.

Rubio, A. O., A. M. M. Stuckert, B. Geralds, R. Nielsen, M. D. MacManes, and K. Summers. 2024. What Makes a Mimic? Orange, Red, and Black Color Production in the Mimic Poison Frog (Ranitomeya imitator). Genome Biol. Evol. 16:evae123.

San-Jose, L. M., F. Granado-Lorencio, B. Sinervo, and P. S. Fitze. 2013. Iridophores and Not Carotenoids Account for Chromatic Variation of Carotenoid-Based Coloration in Common Lizards (Lacerta vivipara). Am. Nat. 181:396–409. The University of Chicago Press.

Schmutz, S. M., and T. G. Berryere. 2007. Genes affecting coat colour and pattern in domestic dogs: a review. Anim. Genet. 38:539–549.

Seddon, R. J., and D. K. Hews. 2016. Populations of the Lizard, Sceloporus occidentalis, that Differ in Melanization have Different Rates of Wound Healing. J. Exp. Zool. Part Ecol. Genet. Physiol. 325:491–500.

Stradi, R., G. Celentano, E. Rossi, G. Rovati, and M. Pastore. 1995. Carotenoids in bird plumage—I. The carotenoid pattern in a series of palearctic carduelinae. Comp. Biochem. Physiol. B Biochem. Mol. Biol. 110:131–143.

Taylor, J. D., and M. E. Hadley. 1970. Chromatophores and color change in the lizard, Anolis carolinensis. Z. Für Zellforsch. Mikrosk. Anat. 104:282–294.

Tibbetts, E. A., and J. Dale. 2004. A socially enforced signal of quality in a paper wasp. Nature 432:218–222. Nature Publishing Group.

Wang, C., B. Lu, T. Li, G. Liang, M. Xu, X. Liu, W. Tao, L. Zhou, T. D. Kocher, and D. Wang. 2021. Nile Tilapia: A Model for Studying Teleost Color Patterns. J. Hered. esab018.

Webber, R., G. Webber Barlow, and A. H. Brush. 1973. Pigments of a color polymorphism in a cichlid fish. Comp. Biochem. Physiol. Part B Comp. Biochem. 44:1127–1135.
