# Supplementary material for: Assessing the Association Between Animal Color and Behavior: A Meta‐Analysis of Experimental Studies
Source: Ecol Evol. 2024 Dec 4;14(12):e70655. doi: 10.1002/ece3.70655 (PMC11617328; doi:10.1002/ece3.70655)
Supplement: Supplementary file 3 — Table S2. Table of model DIC values for random‐effects models. [file ECE3-14-e70655-s002.docx]

Supplemental Table S2: Table of Model DIC Values for Random Effects Models.

| **Model** | **Fixed Effects** | **DIC** | **Percent Heterogeneity** | | | **Phylogenetic Signal (H^2^)** |
| --- | --- | --- | --- | --- | --- | --- |
|  |  |  | **Study**  ${\boldsymbol{(}\boldsymbol{I}}_{\boldsymbol{s}}^{\boldsymbol{2}}\boldsymbol{*}\boldsymbol{100}\boldsymbol{)}$ | **Species**  ${\boldsymbol{(}\boldsymbol{I}}_{\boldsymbol{u}}^{\boldsymbol{2}}\boldsymbol{*}\boldsymbol{100}\boldsymbol{)}$ | **Total (Percentage)** |  |
| Random Includes: species, study, weights, and tree | None (Significant intercept value) | -283.425 | 2.569 | 0.731 | 3.300 | 0.211 |
| Random  Includes: species, study, and weights Missing: Tree | None | -282.069 | -- | -- | -- | -- |
| Random  Includes: species, tree, and weights Missing: study | None | -265.548 | -- | -- | -- | -- |
| Random  Includes: study and weights  Missing: species and tree | None | -282.105 | -- | -- | -- | -- |
| Random  Includes: species, tree, and study Missing: weights | None | 175.204 | -- | -- | -- | -- |
