# Supplementary material for: Assessing the Association Between Animal Color and Behavior: A Meta‐Analysis of Experimental Studies
Source: Ecol Evol. 2024 Dec 4;14(12):e70655. doi: 10.1002/ece3.70655 (PMC11617328; doi:10.1002/ece3.70655)
Supplement: Supplementary file 4 — Table S3. Table of Model DIC values for mixed‐effects models. All means and 95% credible intervals are in correlation coefficient values rather than Fisher Z values for interpretability. [file ECE3-14-e70655-s003.docx]

Supplemental Table S3: Table of Model DIC Values for Mixed Effects Models. All means and 95% credible intervals are in correlation coefficient values rather than Fisher Z values for interpretability.

| **Model** | **Fixed Effects** | **DIC** | **Percent Heterogeneity** | | | **Phylogenetic Signal (H^2^)** | **Mean and 95% Credible Interval** |
| --- | --- | --- | --- | --- | --- | --- | --- |
|  |  |  | **Study**  ${\boldsymbol{(}\boldsymbol{I}}_{\boldsymbol{s}}^{\boldsymbol{2}}\boldsymbol{*}\boldsymbol{100}\boldsymbol{)}$ | **Species**  ${\boldsymbol{(}\boldsymbol{I}}_{\boldsymbol{u}}^{\boldsymbol{2}}\boldsymbol{*}\boldsymbol{100}\boldsymbol{)}$ | **Total (%)** |  |  |
| Mixed | Color Class (full data) | -279.377 | 2.598 | 0.976 | 3.574 | 0.261 | Mean: 0.255  (-0.029, 0.511) |
| Mixed | Color Class (unknown recoded as eumelanin) | -279.590 | 2.524 | 0.940 | 3.464 | 0.259 | Mean: 0.258  (-0.008, 0.499) |
| Mixed | Aggression Measure | -282.652 | 2.598 | 0.607 | 3.205 | 0.180 | Mean: 0.242  (0.040, 0.437)  Direct: 0.295, (0.102, 0.488)  Indirect: 0.19,  (-0.019, 0.386) |
| Mixed | Age Controlled | -281.258 | -- | -- | -- | -- | -- |
| Mixed | Plasticity | -281.700 | -- | -- | -- | -- | -- |
| Mixed | Sex | -280.090 | -- | -- | -- | -- | -- |
| Mixed | Vert/Invert | -280.818 | -- | -- | -- | -- | -- |
| Mixed | Location | -280.487 | -- | -- | -- | -- | -- |
| Mixed | Season | -280.049 | -- | -- | -- | -- | -- |
| Mixed | Life Stage | -279.958 | -- | -- | -- | -- | -- |
| Mixed | Geography | -275.946 | -- | -- | -- | -- | -- |
| Mixed | Social Rank | -276.892 | -- | -- | -- | -- | -- |
| Mixed | Obs vs Exp | -281.522 | -- | -- | -- | -- | -- |
| Mixed | Condition | -280.576 | -- | -- | -- | -- | -- |
| Mixed | Plasticity and Vert/Invert | -281.917 | -- | -- | -- | -- | -- |
| Mixed | Color Class and Life Stage | -277.825 | -- | -- | -- | -- | -- |
| Mixed | Plasticity and Life Stage | -279.764 | -- | -- | -- | -- | -- |
| Mixed | Sex and Life Stage | -278.740 | -- | -- | -- | -- | -- |
| Mixed | Vert/Invert and Life Stage | -279.467 | -- | -- | -- | -- | -- |
| Mixed | Location and Life Stage | -279.210 | -- | -- | -- | -- | -- |
| Mixed | Season and Life Stage | -279.615 | -- | -- | -- | -- | -- |
| Mixed | Plasticity and Sex | -280.470 | -- | -- | -- | -- | -- |
| Mixed | Color Class and Vert/Invert | -278.274 | -- | -- | -- | -- | -- |
| Mixed | Color Class and Location | -278.667 | -- | -- | -- | -- | -- |
| Mixed | Color Class and Season | -278.971 | -- | -- | -- | -- | -- |
| Mixed | Color Class and Plasticity | -280.078 | -- | -- | -- | -- | -- |
| Mixed | Color Class and Sex | -277.890 | -- | -- | -- | -- | -- |
| Mixed | Plasticity and Location | -281.806 | -- | -- | -- | -- | -- |
| Mixed | Plasticity and Season | -279.552 | -- | -- | -- | -- | -- |
| Mixed | Vert/Invert and Sex | -278.817 | -- | -- | -- | -- | -- |
| Mixed | Sex and Location | -279.626 | -- | -- | -- | -- | -- |
| Mixed | Sex and Season | -278.654 | -- | -- | -- | -- | -- |
| Mixed | Vert/Invert and Location | -280.372 | -- | -- | -- | -- | -- |
| Mixed | Vert/Invert and Season | -279.938 | -- | -- | -- | -- | -- |
| Mixed | Location and Season | -280.335 | -- | -- | -- | -- | -- |
| Mixed | Color Class and Aggression Measure | -280.315 | -- | -- | -- | -- | -- |
| Mixed | Color Class and Geography | -274.216 | -- | -- | -- | -- | -- |
| Mixed | Color Class and Social Rank | -275.293 | -- | -- | -- | -- | -- |
| Mixed | Color Class and Obs. vs. Exp. | -279.043 | -- | -- | -- | -- | -- |
| Mixed | Color Class and Condition | -279.001 | -- | -- | -- | -- | -- |
| Mixed | Aggression Measured and Plasticity | -281.928 | -- | -- | -- | -- | -- |
| Mixed | Aggression Measure and Sex | -280.215 | -- | -- | -- | -- | -- |
| Mixed | Aggression Measure and Vert/Invert | -281.282 | -- | -- | -- | -- | -- |
| Mixed | Aggression Measure and Location | -281.422 | -- | -- | -- | -- | -- |
| Mixed | Aggression Measure and Season | -281.225 | -- | -- | -- | -- | -- |
| Mixed | Aggression Measure and Age | -281.190 | -- | -- | -- | -- | -- |
| Mixed | Aggression Measure and Geography | -276.214 | -- | -- | -- | -- | -- |
| Mixed | Aggression Measure and Social Rank | -277.855 | -- | -- | -- | -- | -- |
| Mixed | Aggression Measure and Obs. vs. Exp | -281.965 | -- | -- | -- | -- | -- |
| Mixed | Aggression Measure and Condition | -281.088 | -- | -- | -- | -- | -- |
| Mixed | Plasticity and Geography | -275.768 | -- | -- | -- | -- | -- |
| Mixed | Plasticity and Social Rank | -278.233 | -- | -- | -- | -- | -- |
| Mixed | Plasticity and Obs. vs. Exp | -281.718 | -- | -- | -- | -- | -- |
| Mixed | Plasticity and Condition | -281.346 | -- | -- | -- | -- | -- |
| Mixed | Sex and Geography | -276.291 | -- | -- | -- | -- | -- |
| Mixed | Sex and Social Rank | -277.444 | -- | -- | -- | -- | -- |
| Mixed | Sex and Obs. vs. Exp | -279.730 | -- | -- | -- | -- | -- |
| Mixed | Sex and Condition | -280.189 | -- | -- | -- | -- | -- |
| Mixed | Vert/Invert and Geography | -275.218 | -- | -- | -- | -- | -- |
| Mixed | Vert/Invert and Social Rank | -276.804 | -- | -- | -- | -- | -- |
| Mixed | Vert/Invert and Obs. vs. Exp | -280.569 | -- | -- | -- | -- | -- |
| Mixed | Vert/Invert and Condition | -280.467 | -- | -- | -- | -- | -- |
| Mixed | Location and Geography | -274.319 | -- | -- | -- | -- | -- |
| Mixed | Location and Social Rank | -276.936 | -- | -- | -- | -- | -- |
| Mixed | Location and Obs. vs. Exp | -280.214 | -- | -- | -- | -- | -- |
| Mixed | Location and Condition | -279.733 | -- | -- | -- | -- | -- |
| Mixed | Season and Geography | -274.683 | -- | -- | -- | -- | -- |
| Mixed | Season and Social Rank | -275.466 | -- | -- | -- | -- | -- |
| Mixed | Season and Obs. vs. Exp | -280.537 | -- | -- | -- | -- | -- |
| Mixed | Season and Condition | -274.647 | -- | -- | -- | -- | -- |
| Mixed | Life Stage and Geography | -275.420 | -- | -- | -- | -- | -- |
| Mixed | Life Stage and Social Rank | -276.195 | -- | -- | -- | -- | -- |
| Mixed | Life Stage and Obs. vs. Exp | -280.341 | -- | -- | -- | -- | -- |
| Mixed | Life Stage and Condition | -280.625 | -- | -- | -- | -- | -- |
| Mixed | Geography and Social Rank | -273.786 | -- | -- | -- | -- | -- |
| Mixed | Geography and Obs. vs. Exp | -275.708 | -- | -- | -- | -- | -- |
| Mixed | Geography and Condition | -275.491 | -- | -- | -- | -- | -- |
| Mixed | Social Rank and Obs. vs. Exp | -277.373 | -- | -- | -- | -- | -- |
| Mixed | Social Rank and Condition | -276.656 | -- | -- | -- | -- | -- |
| Mixed | Obs. vs. Exp and Condition | -280.562 | -- | -- | -- | -- | -- |
| Mixed | Age Controlled and Color Class | -278.379 | -- | -- | -- | -- | -- |
| Mixed | Age Controlled and Agg Measure | -281.636 | -- | -- | -- | -- | -- |
| Mixed | Age Controlled and Plasticity | -281.765 | -- | -- | -- | -- | -- |
| Mixed | Age Controlled and Sex | -278.395 | -- | -- | -- | -- | -- |
| Mixed | Age Controlled and Vert/Invert | -280.387 | -- | -- | -- | -- | -- |
| Mixed | Age Controlled and Location | -279.004 | -- | -- | -- | -- | -- |
| Mixed | Age Controlled and Seasonality | -279.967 | -- | -- | -- | -- | -- |
| Mixed | Age Controlled and Life Stage | -280.344 | -- | -- | -- | -- | -- |
| Mixed | Age Controlled and Geographic | -274.626 | -- | -- | -- | -- | -- |
| Mixed | Age Controlled and Social Rank | -276.875 | -- | -- | -- | -- | -- |
| Mixed | Age Controlled and Obs. vs. Exp. | -280.829 | -- | -- | -- | -- | -- |
| Mixed | Age Controlled and Condition | -280.034 | -- | -- | -- | -- | -- |
| Mixed | Color, Plasticity, and Color by Plasticity | -275.966 | -- | -- | -- | -- | -- |
| Mixed | Color, Sex, and Color by Sex | -274.065 | -- | -- | -- | -- | -- |
| Mixed | Sex, Plasticity, and Sex by Plasticity | -277.397 | -- | -- | -- | -- | -- |
