# Supplementary material for: Assessing the Association Between Animal Color and Behavior: A Meta‐Analysis of Experimental Studies
Source: Ecol Evol. 2024 Dec 4;14(12):e70655. doi: 10.1002/ece3.70655 (PMC11617328; doi:10.1002/ece3.70655)
Supplement: Supplementary file 5 — Table S4. Table of model DIC values for subset models. Random‐effects–only models of different data subsets are listed first in the table, followed by corresponding models that included the color moderator. All means and 95% credible intervals are correlation coefficients rather than Fisher Z values and are corrected for publication bias when necessary. [file ECE3-14-e70655-s001.docx]

Supplemental Table S4: Table of Model DIC Values for Subset Models. Random-effects-only models of different data subsets are listed first in the Table, followed by corresponding models that included the color moderator. All means and 95% credible intervals are correlation coefficients rather than Fisher Z values and corrected for publication bias when necessary.

| **Model** | **Fixed Effects** | **DIC** | **Number of Papers** | **Number of Effect Sizes** | **Effect Sizes per Color Class** | **Mean and 95% Credible Interval** |
| --- | --- | --- | --- | --- | --- | --- |
| Random Includes: species, study, weights, and tree | None  Subset by: Controlled Social Rank | -221.320 | 58 | 133 | 40 carotenoid, 47 eumelanin, 28 pheomelanin, 1 pteridine, 6 structural, and 11 unknown | Mean: 0.193  (0.047, 0.336) |
| Random Includes: species, study, weights, and tree | None  Subset by: Controlled Condition | -127.049 | 59 | 112 | 39 carotenoid, 57 eumelanin, 1 pheomelanin, 1 pteridine, 6 structural, and 8 unknown | Mean: 0.115  (-0.138, 0.408) |
| Random  Includes: species, study, weights, and tree | None  Subset by: Controlled Age | 14.183 | 16 | 25 | 8 carotenoid, 13 eumelanin, 2 pheomelanin, 1 structural, and 1 unknown | Mean: 0.260  (-0.430, 1.078) |
| Random Includes: species, study, weights, and tree | None  Subset by: Controlled Social Rank and Condition | -114.390 | 45 | 87 | 31 carotenoid, 41 eumelanin, 1 pheomelanin, 1 pteridine, 6 structural, and 7 unknown | Mean: 0.163  (0.012, 0.331) |
| Random  Includes: species, study, weights, and tree | None  Subset by: Controlled Age, Social Rank, and Condition | -4.436 | 9 | 15 | 8 carotenoid, 6 eumelanin, and 1 structural | Mean: 0.177  (-0.181, 0.523) |
| Mixed | Color Class  Subset by: Controlled Social Rank | -214.482 | 58 | 133 | 40 carotenoid, 47 eumelanin, 28 pheomelanin, 1 pteridine, 6 structural, and 11 unknown | Mean: 0.203,  (-0.041, 0.424) |
| Mixed | Color Class  Subset by: Controlled Condition | -123.755 | 59 | 112 | 39 carotenoid, 57 eumelanin, 1 pheomelanin, 1 pteridine, 6 structural, and 8 unknown | Mean: 0.173  (-0.185, 0.514) |
| Mixed | Color Class Subset by: Controlled Age | 12.208 | 16 | 25 | 8 carotenoid, 13 eumelanin, 2 pheomelanin, 1 structural, and 1 unknown | Mean: 0.289  (-1.225, 1.844) |
| Mixed | Color Class  Subset by: Controlled Social Rank and Condition | -111.656 | 45 | 87 | 31 carotenoid, 41 eumelanin, 1 pheomelanin, 1 pteridine, 6 structural, and 7 unknown | Mean: 0.158  (-0.081, 0.388) |
